# Fecal tryptophan metabolite profiling in newborns in relation to microbiota and antibiotic treatment

**DOI:** 10.1007/s00253-024-13339-4

**Published:** 2024-11-05

**Authors:** Anne-Christine Aust, Veronika Vidova, Katerina Coufalikova, Sona Smetanova, Kristyna Kozeluhova, Lenka Micenkova, Petra Videnska, Stanislav Smatana, Eva Budinska, Ivo Borek, Petr Janku, Jana Klanova, Zdenek Spacil, Vojtech Thon

**Affiliations:** 1 RECETOX, Faculty of Science, Masaryk University, Kamenice 753/5, pavilion D29/1S101, 625 00 Brno, Czech Republic; 2 Department of Neonatology, University Hospital Brno, Brno, Czech Republic; 3 Clinic of Gynecology and Obstetrics, University Hospital Brno, Brno, Czech Republic

**Keywords:** Stool, Microbiome, Tryptophan catabolites, Kynurenine, Vaginal delivery, Caesarean delivery

## Abstract

**Abstract:**

In the first days of life, the newborns’ intestinal microbiota develops simultaneously with the intestinal gut barrier and follows intestinal immunity. The mode of delivery shows significant impact on microbial development and, thus, the initiation of the tryptophan catabolism pathway. Further antibiotics (ATB) treatment of mothers before or during delivery affects the microbial and tryptophan metabolite composition of stool of the caesarean- and vaginal-delivered newborns. The determination of microbiome and levels of tryptophan microbial metabolites in meconium and stool can characterize intestinal colonization of a newborn. From 134 samples from the Central European Longitudinal Studies of Parents and Children: The Next Generation (CELSPAC: TNG) cohort study, 16S rRNA gene sequencing was performed, and microbial tryptophan metabolites were quantified using ultra-high-performance liquid chromatography with triple-quadrupole mass spectrometry. Microbial diversity and concentrations of tryptophan metabolites were significantly higher in stool compared to meconium. Treatment of mothers with ATB before or during delivery affects metabolite composition and microbial diversity in stool of vaginal- and caesarean-delivered newborns. Correlation of microbial and metabolite composition shows significant positive correlations of indol-3-lactic acid, *N*-acetyl-tryptophan and indol-3-acetic acid with *Bifidobacterium*, *Bacteroides* and *Peptoclostridium*. The positive effect of vaginal delivery on newborns’ microbiome development is degraded when mother is treated with ATB before or during delivery.

**Key points:**

• *Antibiotic treatment diminishes the positive effects of vaginal delivery.*

• *Antibiotic treatment affects metabolite and microbial composition in newborns.*

• *Bifidobacterium and Peptoclostridium could be the producer of indole-lactic acid.*

**Supplementary Information:**

The online version contains supplementary material available at 10.1007/s00253-024-13339-4.

## Introduction

Newborns get in touch with various impacts influencing their own microbiome. Childbirth and the first days of life significantly affect microbial development of a newborn. The unique bacterial composition is appropriate for vaginal (VD) or caesarean (CD) delivery mode. In VD, the leading bacterial phylum reflects the oral microbiome (Dominguez-Bello et al. 2010), and gut bacteria are transferred from the mother to the child (Neu and Rushing 2011). The vagina as an environment is rich in diverse species and beneficial for the newborn, which is essential for establishing the immune initiative system (Shao et al. 2019). In CD, the bacterial composition differs (Laforest-Lapointe and Arrieta 2017). The microbiome reflects the composition of the hospital environment’s surroundings, which has less diversity in the bacterial species (Shao et al. 2019). Meconium is passed prior to stool and differs in appearance and composition (Gosalbes et al. 2013). According to Gosalbes et al. (2013), two clusters of microbiota composition can be distinguished in meconium. The first cluster is dominated by *Enterobacteriaceae*, with *Escherichia-Shigella* as the most abundant genus. The second cluster is dominated by *Firmicutes* and lactic acid bacteria families, *Leuconostocaceae*, *Enterococcaceae* and *Streptococcaceae*. The most abundant genera in the second cluster are *Leuconostoc*, *Enterococcus*, *Lactococcus*, *Staphylococcus* and *Streptococcus* (Gosalbes et al. 2013). The microbiota of meconium differed as well in the type of delivery. CD samples belonged to cluster 2 in Gosalbes’ study. VD meconium samples were equally distributed between two clusters of bacterial composition (Gosalbes et al. 2013). Stool samples have been found to differ between the mode of delivery (Brazier et al. 2017). VD newborn stool contained abundant genera of *Bacteroides* and *Collinsella*, while in CD newborn stool predominated *Sarcina* and *Klebsiella* genera (Brazier et al. 2017). The meconium and stool samples were investigated for the harbored microbiome because it reflects the gut bacteria diversity for 67.7% (Zierer et al. 2018; Inchingolo et al. 2024).

Furthermore, the microbial composition in CD newborns is led by opportunistic pathogens, which can carry plasmids for antibiotic resistance (Shao et al. 2019). CD is usually accompanied by antibiotics (ATB) intake due to caesarean prophylaxis. In VD, the ATB are given immediately prior to delivery when the *Streptococcus* B is indicated in the vagina microbiome to prevent neonatal sepsis (Verani et al. 2010; De Tejada 2014). The ATB intake influences the microbiome composition in the intestine (Elvers et al. 2020). Further, ATB intake significantly decreased the effect on the maturation of the immune function in the small intestine and colon (Schumann et al. 2005).

Tryptophan (TRP) metabolites have an essential role in keeping homeostasis in the intestine (Roager and Licht 2018), counteract inflammation (Roager and Licht 2018) and protect from oxidative damage in the brain by passing the blood–brain barrier (Poeggeler et al. 1999; Desbonnet et al. 2008). Furthermore, TRP and its metabolites bind to the aryl-hydrocarbon receptor (AhR), which is expressed by many immune cells. Through binding of AhR, the metabolites have a pleiotropic effect on many immune cell types, including dendritic cells and macrophages, and they contribute to an anti-inflammatory environment in the gut (Scott et al. 2020). Thus, in this study, we have quantified tryptophan and TRP metabolites: anthranilate (ATA), indole-3-acetic acid (IAA), indole-3-aldehyde (IAld), indole-3-lactic acid (ILA), *N*-acetyl-tryptophan (NAceTRP) and kynurenine (KYN) that are formed by bacteria in the newborn stool.

The immunoglobulin A (IGHA) is an essential key player for mucosal immune system development in early life (Gleeson and Cripps 2004). Newborns start to produce their own IGHA. Normal ranges of IGHA in childhood are reached by the end of the first year (Rule et al. 1971). The IGHA is a significant part of breastmilk and is delivered to the infant via breastfeeding. Thus, IGHA is not present in the newborn’s meconium samples, but it is detected in the infant’s stool as passed breast milk (Lisowska-Myjak and Pachecka 2007). Through this fact, IGHA can be used to differentiate between meconium and stool (Meyer et al. 2019).

In our previous work, we quantified TRP metabolites systemically in neonatal dried blood spots (DBS) on day 2 of the newborn’s life (Aust et al. 2021). In this follow-up study, we developed a mass spectrometry method for the in situ quantification of TRP and its metabolites in newborns’ meconium and stool. Therefore, we quantified TRP, ATA, IAA, IAld, ILA, NAceTRP and KYN in 134 meconium and stool samples. The bacterial composition of all meconium and stool samples was assessed using 16S rRNA gene sequencing. We investigated the relationship between the gut microbiota and its produced TRP metabolites in the context of gut barrier development in meconium and stool. We studied the effect of delivery mode and ATB treatment on newborns’ microbiome development and association with TRP metabolite production. The differentiation could show differences in its outcome for shaping the immune system in the first three days of life.

## Material and methods

### Study design

Infants’ meconium and stool samples were collected between day 1 and day 4 from birth from 134 neonates (20 delivered via caesarean section and 114 delivered vaginally) at the University Hospital Brno within the CELSPAC: TNG cohort study (Central European Longitudinal Study of Parents and Children: The Next Generation). The CELSPAC: TNG study is designed as a new prospective birth cohort that will follow up on 3000 children from their prenatal period to adolescence to assess multiple factors potentially affecting children’s health. The Multicentre and Local Ethical Committee of University Hospital Brno, the Czech Republic, approved this study (No. 20140409–01). Participating mothers signed the informed consent form on behalf of their newborn baby.

Flocked swabs (Copan, Brescia, Italy) were used for sample collection, and all samples were stored at − 80 °C. Characteristics of individual neonates, including gestational age, delivery mode, sex, birth weight, birth length, Apgar score and medication, are shown in Supplementary Table S1.

### DNA isolation from meconium/stool samples, PCR amplification and *sequencing* of *16S rRNA gene*

DNA isolation of 134 meconium and stool samples was performed using a PowerLyzer® PowerSoil® DNA Isolation kit (QIAGEN, Hilden, Germany), according to the manufacturer’s protocol. Isolated DNA was used as a template in PCR reactions targeting the hypervariable V4 region (EMP 515–806) of the bacterial 16S rRNA gene according to the 16S Metagenomic Sequencing Library Preparation protocol (Illumina, San Diego, California, USA) (Supplementary Table S2). Sequencing was performed using MiSeq Reagent Kits v2 on a MiSeq 2000 sequencer according to the manufacturer’s instructions (Illumina, San Diego, California, USA).

### Bioinformatics analysis

The raw sequence reads were pre-processed by the following pipeline. The first step of the pipeline was demultiplexing of reads in sequencing pools into individual samples. The next step in the pipeline was trimming the low-quality end of each read.

Both demultiplexing and length filtering were performed by an in-house tool written in Python 3. Forward and reverse reads were denoised using the DADA2 amplicon denoising R package (Callahan et al. 2016). Following denoising, the forward and reverse reads were joined using the fastq-join read joining utility (Aronesty 2013). Finally, chimeric sequences were removed from the joined reads using the removeBimera function of the DADA2 R package. Taxonomy was determined using the usearch-consensus algorithm from the microbiome analysis toolkit QIIME (v 1.9.1) (Caporaso et al. 2011). For each input sequence, the three closest organisms were found in the Silva v. 123 reference database (Quast et al. 2013). The sequenced raw data were deposited under the accession number EBI-ENA BioProject PRJEB79120.

### Chemicals and reagents

Metabolomics isotope-labelled standards on carbon [13C6], [13C11], nitrogen [15N2], or deuterium [2D4] were used. Isotope-labelled [13C6] indole-3-acetic acid (cat. #0317333) was purchased from OlChemIm s.r.o. (Olomouc, Czech Republic). Isotope-labelled [13C11] [15N2] l-tryptophan (cat. #574597), purity ≥ 98%, was purchased from Sigma-Aldrich (St. Louis, Missouri, USA). Isotope-labelled [2D4] l-kynurenine (cat. #DLM-7842-PK), purity of 95%, was purchased from Cambridge Isotope Laboratories, Inc. (Tewksbury, Massachusetts, USA). Isotope-labelled [13C6] anthranilic acid (cat. #PR-24225), purity 99%, was purchased from Sigma-Aldrich (St. Louis, Missouri, USA). Liquid chromatography-mass spectrometry (LC–MS) grade acetonitrile (ACN) (cat. #0013687802BS) and isopropanol (cat. #0016267802BS) were purchased from Biosolv BV. Formic acid (FA) for mass spectrometry (cat. #94318) was purchased from Sigma-Aldrich (St. Louis, Missouri, USA). Deionized water was produced using Millipore Simplicity 185 ultrapure water system (Merck Millipore corp. Billerica, Massachusetts, USA).

The chemical standard of l-tryptophan (cat. #51145) (TraceCERT®), *N*-acetyl-tryptophan (cat. #PHR1177), indole-3-acetate (cat. #45533), purity 98%, l-kynurenine (cat. #K8625), purity ≥ 98%, were purchased from Sigma-Aldrich (St. Louis, MO, USA). The chemical standard indole-3-carboxaldehyde (cat. #A15330), purity 99%, was purchased from Alfa Aesar (Haverhill, MA, USA). The standard of indole-3-lactic acid (≥ 97%) (cat. #SC-255130), purity ≥ 97%, was purchased from Santa Cruz Biotechnology (Dallas, TX, USA).

### Proteomics analysis

Target and total protein analysis of meconium and stool samples from 134 newborns was previously described by Vidova et al. (2021). The protein IGHA 1 + 2 can be found under the accession number P01876 + P01877 (Vidova et al. 2021).

### Metabolite extraction protocol

Prior to extraction, samples were taken out of the − 80 °C freezer and left at lab temperature for 30 min. One millilitre of 80% isopropanol was added into a vial with a stool swab and extracted in an orbital shaker (5 min, 1600 rpm). Then, the sample was centrifuged (2 min, 12,000 × *g*). Fifty microlitres of the extract was pipetted into a new vial and dried out in a vacuum concentrator for 30 min (Savant SPD121 P SpeedVac, Thermo Fisher, Waltham, MA, USA). The dried extract was resolved with a solution of 5% isopropanol containing isotopically labelled standards with a concentration of 50 nM [13C6] ATA, 200 nM [13C6] IAA, 50 nM [2D4] KYN and 2000 nM [13C11] [15N2] TRP. Dissolution was supported using ultrasonic bath (1 min, 37 °C).

### Metabolomics UHPLC-SRM assay

Samples were analysed using ultra-high-performance liquid chromatography (UHPLC) and tandem mass spectrometry (MS/MS) using selected reaction monitoring (SRM) mode. Samples were injected (2 µL) on the UHPLC system (1260 Infinity II) from Agilent Technologies (Santa Clara, California, USA) equipped with Acquity UHPLC CSH C18 column (100 × 2.1 mm, 1.7 μm) and VanGuard ACQUISITY UPLC CSH C18 (5 × 2.1 mm; 1.7 μm) pre-column from Waters (Waters GmbH, Eschborn, Germany). The column was thermostated at 40 °C. The following mobile phases were used: A (0.1% FA in water) and B (0.1% FA in 95% ACN) at a 0.3 mL/min flow rate. The gradient elution program (0–14.0 min) with equilibration step (12.0–14.0 min) was set as follows: 0.0 min 5% B; 5 min 10% B; 10 min 95% B; 11.99 min 95% B; 12 min 5% B; 14 min 5% B. The UPLC system was coupled to a triple quadrupole mass spectrometer Agilent 6495A (Agilent Technologies, Santa Clara, CA, USA) with an electrospray source operated in positive ion mode with a capillary voltage of 3500 V. Additional ion source parameters were the following: gas flow rate 15 L/min at 160 °C, sheath gas pressure 25 PSI at 250 °C and nozzle voltage 500 V. Optimizer software (Agilent Technologies, Santa Clara, CA, USA) was used for generation SRM libraries in positive ion detection mode from a standard solution of an individual metabolite standard. For metabolite identification, 2–6 SRM qualifier transitions were monitored per metabolite, and the single best-performing SRM transition was used for quantification. The set of SRM transitions had been taken for isotope-labelled metabolite standards, and non-labelled metabolites, i.e., 23 transitions, were monitored per analysis (Suppl. Figure S1, Suppl. Table S3).

### Method validation and reproducibility

The protein assay validation was reported previously (Vidova et al. 2021). A pool of extract from 134 meconium and stool samples was used for method validation. The method was validated using matrix matched calibration curves to determine the linearity range, coefficient of determination (*R*^2^), limit of detection (LOD) and limit of quantification (LOQ). The dilution series consisted of six concentration levels and was measured at 3–6 technical replicates (Suppl. Figure S2, S3, S4 and S5). LOD and LOQ were determined for isotopically labelled ATA, IAA, KYN and TRP. The LOD and LOQ were calculated of the calibration curve slope and the labelled metabolites’ response factors (Suppl. Equation 1–4). The LOD and LOQ for the quantified metabolites are listed in Suppl. Table S4. The linearity range is shown in Suppl. Table S5. For interday and intraday reproducibility, metabolite extracts were processed and analysed for three consecutive days at six replicates daily. The interday reproducibility ranged from CV 8 up to 20%. The intraday reproducibility ranged from CV 11 up to 22% (data not shown).

### Tryptophan metabolite quantification

The concentrations of ATA, IAA, KYN and TRP were determined in stool extracts using internal standardization with isotope-labelled standards at concentrations of 50, 200, 50 and 1000 nM, respectively. The concentration of IAld, ILA and NAaceTRP was determined using the response factor (RF). The calculation is based on the concentration of isotope-labelled metabolites added to stool extracts (*CIS*), the measured response (integrated peak area) of isotope-labelled metabolite standard (*AIS*) and the response (integrated peak area) of the metabolite (*Amet*) (Eq. 1). The concentration of IAld, ILA and NAaceTRP was corrected with the response factor (Eq. 2), determined in a conventional manner (Vidova and Spacil 2017) (Eq. 3).

1 $$cmet=\left(\frac{CIS}{AIS}xAmet\right)$$

2 $$\mathrm{cmet}=\left(\frac{CIS}{AIS}xAmet\right):RF$$

3 $$RF=\left(\frac{Amet}{AIS}x\frac{CIS}{Cmet}\right)$$

### Statistical analysis

Metabolite and protein concentrations were log-transformed before statistical analysis, and values below LOQ and LOD were substituted with √2 /2 *LOQ and √2 /2 *LOD, respectively. Only analytes with < 25% substitution were used for the multidimensional statistical analysis (PCA, hierarchical clustering) as continuous quantitative variables (Antweiler 2015; Hazra and Gogtay 2017). Due to a high percentage of values below LOQ, *ATA*, *IAA*, *KYN* and *NAceTRP* were categorized and used only as additional variables with three categories—“*below LOD*”, “*below LOQ*” and “*above LOQ*”. For one-dimensional analysis (hypothesis tests, correlation), these analytes were involved uncategorized using non-parametric methods.

Differences in concentrations of analytes between various groups of samples (type of delivery, ATB treatment, stool category) were tested using a parametric *t*-test for the metabolites TRP, IAld and ILA. For the metabolites ATA, IAA, KYN and NAceTRP, the significance was tested with a nonparametric Mann–Whitney *U* test. The resulting *p*-values were adjusted for multiple hypothesis testing using the Benjamini–Hochberg procedure. Results were considered significant at false discovery rate (FDR) ≤ 0.05.

Differences in microbiome alpha diversity (estimated by the Shannon diversity index) between various groups of samples were statistically evaluated using Mann–Whitney *U* test.

Statistical analysis of sequencing microbiome data was done on the genus level. Data were treated as compositional and before all statistical analyses were transformed using centred log-ratio (CLR) transformation (Aitchison 1986). All zeroes in the original data were replaced using the count zero multiplicative method (Martín-Fernández et al. 2015). Only taxa with a percentage representation of ≥ 0.3% of minimal sequencing depth in at least three samples were included in the transformation process and additional statistical analysis of the microbiome to avoid high sparsity in data. Mann–Whitney *U* test was used to compare the differences between the groups for each taxa. The resulting *p*-values were adjusted for multiple hypothesis testing using the Benjamini–Hochberg procedure (BH). Results were considered significant at FDR ≤ 0.1.

Differences in global metabolome, proteome and microbiome composition and their variability in samples were studied using principal component analysis (PCA) and heatmaps with hierarchical clustering (Ward method on Euclidean distance was applied to samples and bacteria and distance derived from Pearson correlation to the metabolites and metabolites + bacteria).

Spearman’s rank correlation was performed to examine the relationships among metabolites and bacteria. The resulting *p*-values were adjusted for multiple hypothesis testing using the Benjamini–Hochberg procedure. Results were considered significant at FDR ≤ 0.1.

All statistical analyses were performed in R, version 4.0.3 (R core team, 2020), using additional R packages *ggplot2* (plotting) (Wickham 2009), *nortest* (normality testing) (Gross and Ligges 2015), *gplots* (Warnes et al. 2020), *zCompositions* (zero replacement) (Palarea-Albaladejo and Martín-Fernández 2015), *compositions (*CLR transformation) (Van den Boogaart et al. 2022), *ggpubr* (plotting data) (Kassambara 2020), *ComplexHeatmap* (heatmaps) (Gu et al. 2016), *corrplot* (correlation matrix plot) (Wei and Simko 2017) and *beeswarm* (boxplots) (Eklund 2016).

## Results

The development of early intestinal microbiome and gut mucosal immune system was studied in 134 newborns using metabolomics and microbiome analysis of meconium and stool samples. Proteomics analysis was previously published by Vidova et al. (2021). In this work, proteomics data were used to identify stool and meconium based on the concentration of IGHA and PCA analysis.

### Differentiation between meconium and stool

TRP and TRP metabolites (ATA, IAA, ILA, IAld, KYN and NAceTRP) were analysed in meconium and stool samples from 134 newborns. The samples were collected by hospital personnel 10 to 179 h after delivery and visually evaluated whether the sample was meconium or stool by colour, consistency and odour. All samples were split into two groups, meconium and stool, because of the different properties of these two matrices. We defined meconium and stool on the basis of IGHA concentration (derived from breast milk), since visual assessment by hospital personnel was rather uncertain. Supplementary Figure S6 shows a histogram and density function of IGHA concentration in the stool samples. The red peak indicates meconium, and the green peak indicates stool. A local minimum is 0.04242 which corresponds to the concentration 0.90694 µg/mg IGHA and defines samples with the concentration of IGHA below this minimum as meconium and samples with a concentration above this minimum as stool samples.

Because of the wide time frame when the samples were collected, samples were categorized in into six time frames to visualize how time affects the metabolite composition during the first days of life. Supplementary Figure S7 shows the variability of samples based on the metabolite composition in various times. PCA plot indicates that metabolite composition formatted within 24 h is more altered after 72 h (blue dots) after delivery. It is assumed that meconium is passed mostly within 24 h and after 72 h, the only stool is passed. Between 24 and 72 h, the combination of both is passed. Identification of meconium and stool based on the time caused elimination of all samples that were collected between 24 and 72 h and reduction of the data set.

**Fig. 1:**
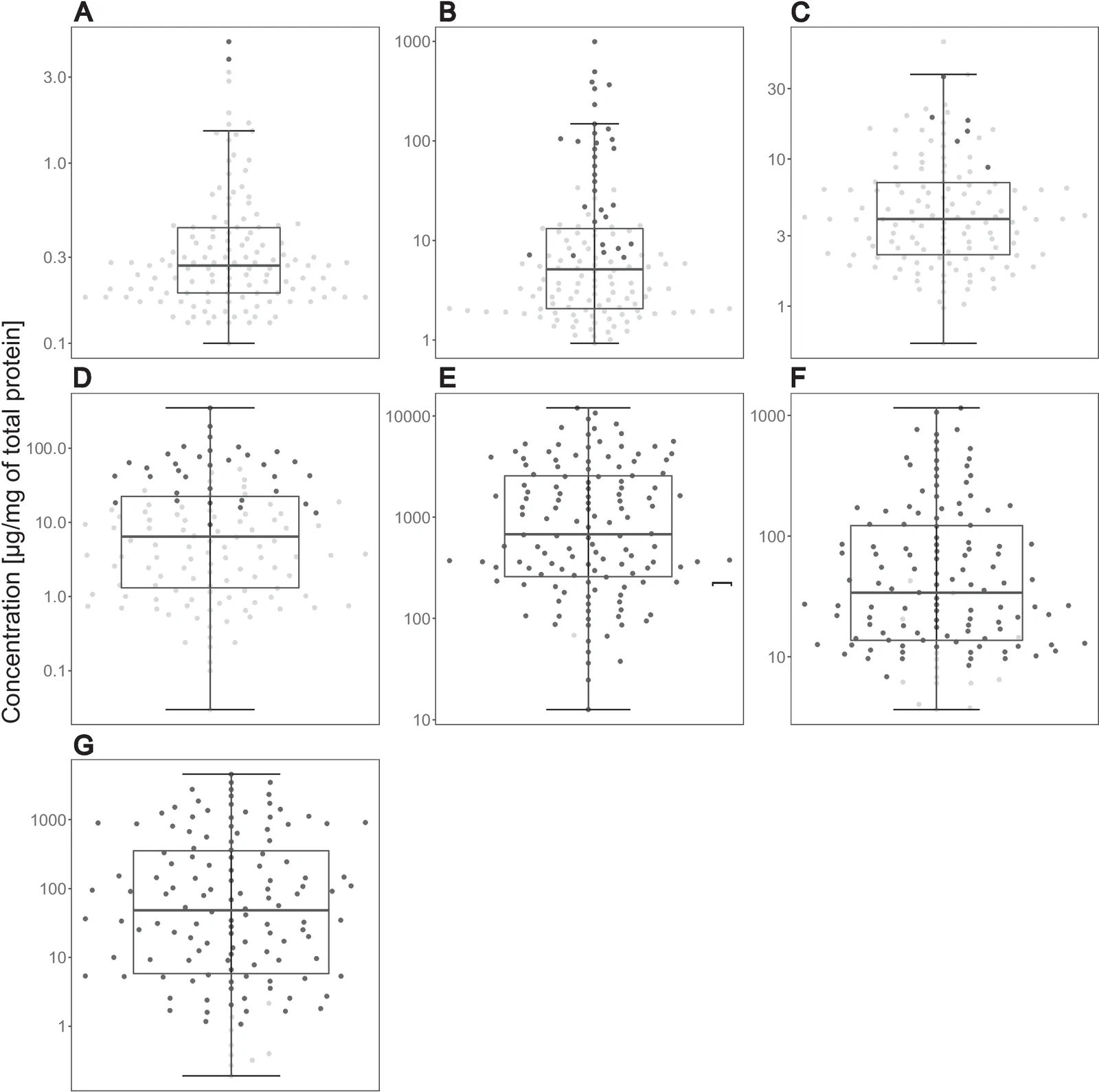
Concentrations of metabolites in meconium and stool samples from 134 newborns. Black dots represent concentrations of metabolites > LOQ. Grey dots represent concentrations < LOQ. Boxplots represent concentrations of ATA (**A**), IAA (**B**), KYN (**C**), NAceTRP (**D**), TRP (**E**), IAld (**F**), and ILA (**G**). The box represents the middle 50%, the bold horizontal line inside the box represents the median (Q2), the bottom of the box is at the first quartile (Q1; 25th percentile), the top of the box is at the third quartile (Q3; 75th percentile). Whiskers extend to the smallest and largest values within 1.5 times the IQR (1.5 × IQR) from Q1 and Q3, respectively (IQR = inter-quartile range). Points plotted individually beyond the whiskers represent outliers

**Fig. 2:**
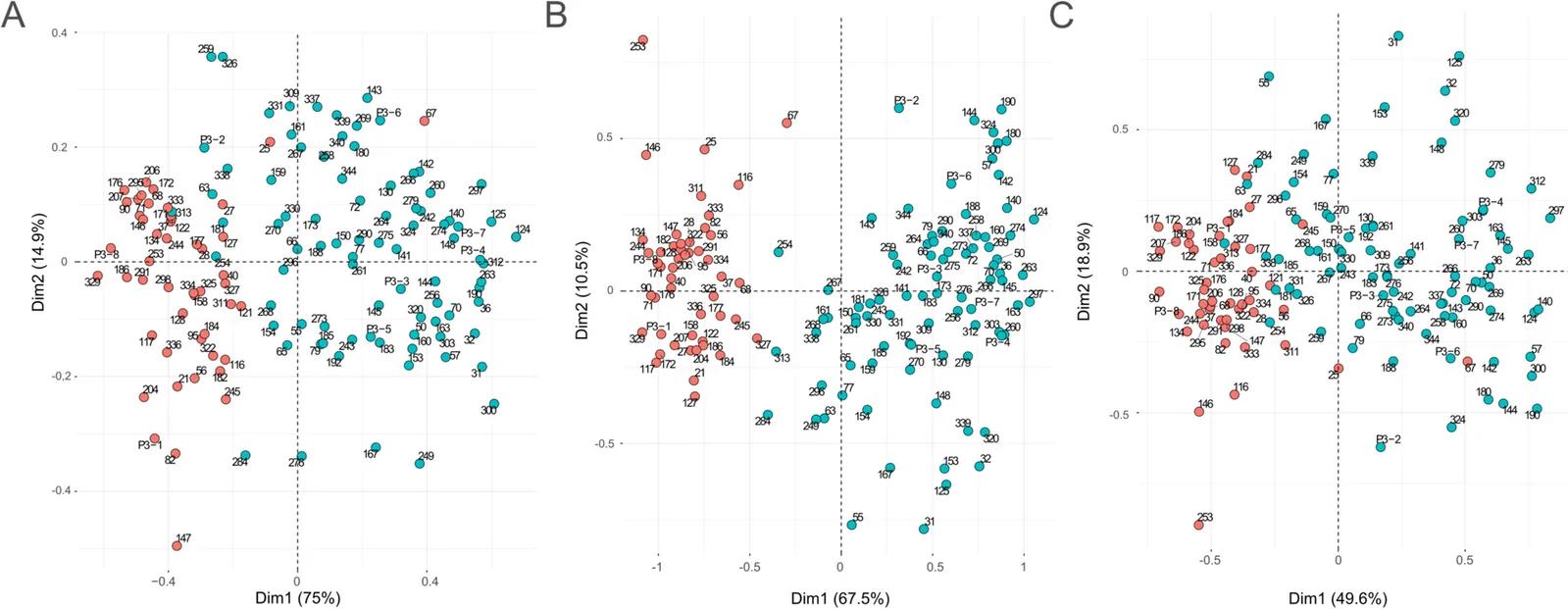
PCA plots of quantified metabolites TRP, ILA, IAld (**A**), metabolites and proteins with IGHA (**B**), metabolites and proteins without IGHA (**C**). Red dots indicate meconium samples, and green dots indicate stool samples identified based on the concentration of IHGA in the sample

The metabolites ATA, IAA, IAld, ILA, KYN, NAceTRP and TRP were measured in meconium and stool samples (Fig. 1). PCA plots of metabolites and protein concentrations in meconium and stool samples are shown in Fig. 2. As indicated in Fig. 2B (metabolites and proteins with IGHA), the meconium and stool samples are differentiated by IGHA. Although PCA plots of metabolites and proteins without IGHA (Fig. 2A, C) did not show such strong separation between meconium and stool, the division is still visible and revealed outlying samples. The outlying samples are 25 and 67, which will be described later.

### Metabolite analysis and 16S rRNA gene sequencing

The definition of the sample allowed us to compare the metabolite and microbiome composition (16S rRNA gene sequencing) in meconium and stool. Concentrations of metabolites ILA, TRP and IAld were significantly higher in stool than in meconium (Supplementary Figure S8). Although most of the concentrations of ATA, IAA, NAceTRP and KYN were below LOQ, the elevated concentration of metabolites in stool is visible (Supplementary Figure S9). Microbiome analysis showed a significantly higher diversity index in stool than in meconium (*p* < 0.0005, data not shown).

**Fig. 3:**
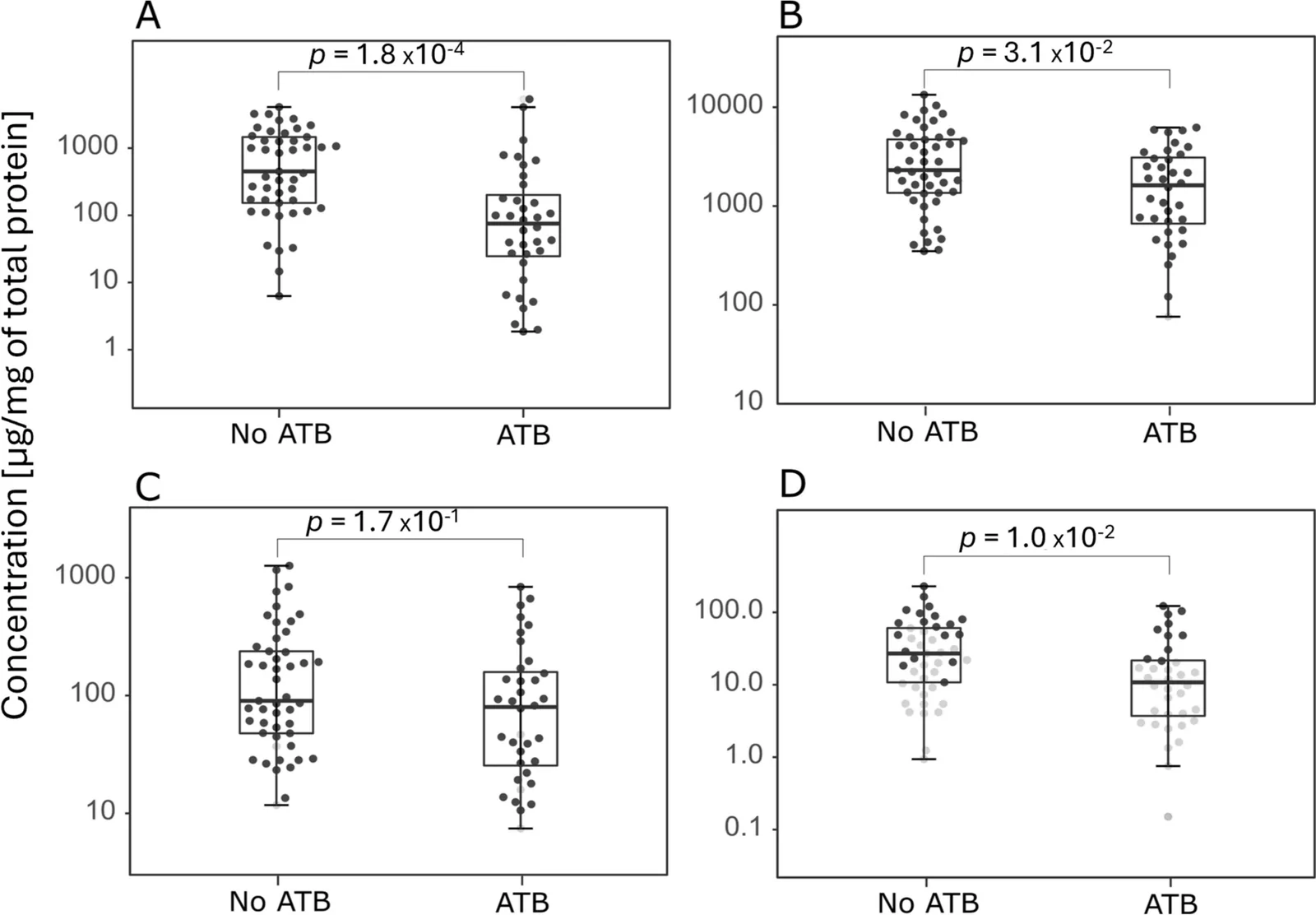
Comparison of metabolite concentrations in stool samples from newborns of ATB-treated mothers during or after delivery. Black dots represent concentrations of metabolites > LOQ. Grey dots represent concentrations < LOQ. Boxplots represent grouped concentrations of ILA (**A**), TRP (**B**), IAld (**C**), and NAceTRP (**D**). Nonparametric Mann–Whitney *U* test was used here. The box represents the middle 50%, the bold horizontal line inside the box represents the median (Q2), the bottom of the box is at the first quartile (Q1; 25th percentile), the top of the box is at the third quartile (Q3; 75th percentile). Whiskers extend to the smallest and largest values within 1.5 times the IQR (1.5 × IQR) from Q1 and Q3, respectively (IQR = inter-quartile range). Points plotted individually beyond the whiskers represent outliers

**Fig. 4:**
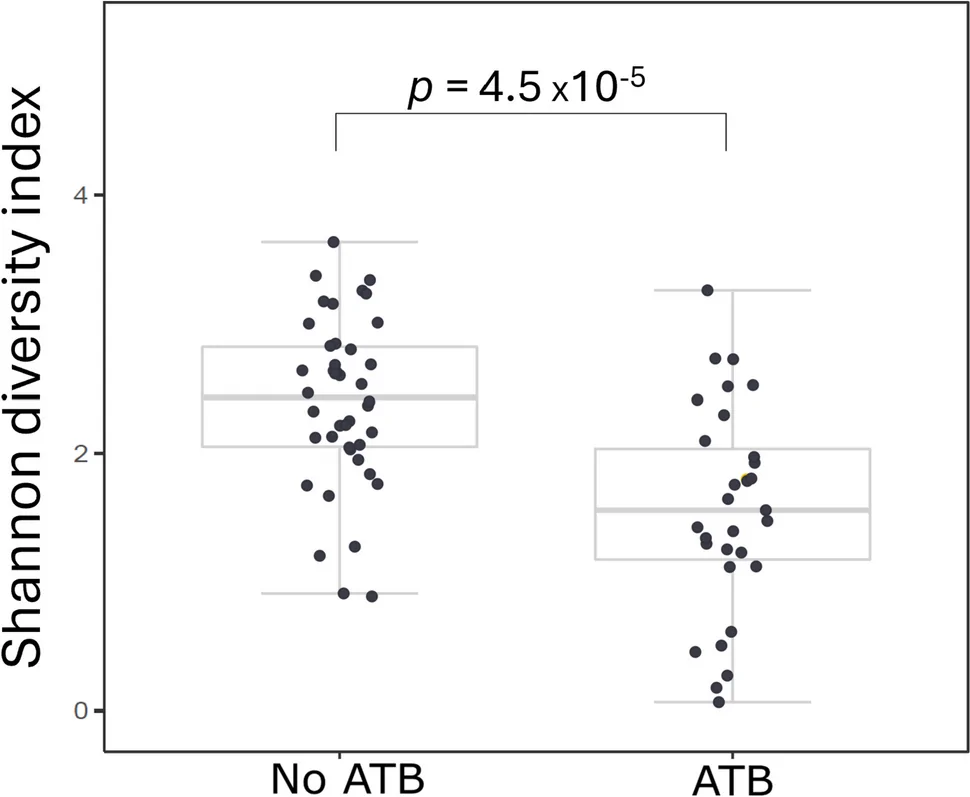
The effect of ATB-treatment of mothers on microbial diversity in the stool of newborns. Comparison of microbial diversity of newborns delivered by ATB treated (left box) and ATB untreated mothers (right box). Nonparametric Mann–Whitney *U* test was used here. The box represents the middle 50%, the bold horizontal line inside the box represents the median (Q2), the bottom of the box is at the first quartile (Q1; 25th percentile), the top of the box is at the third quartile (Q3; 75th percentile). Whiskers extend to the smallest and largest values within 1.5 times the IQR (1.5 × IQR) from Q1 and Q3, respectively (IQR = inter-quartile range). Points plotted individually beyond the whiskers represent outliers

The 51 mothers were treated with beta-lactam antibiotics (ATB) or lincosamide ATB before or during delivery. The concentration of ILA, TRP and NAceTRP in newborns stool of ATB-treated mothers was significantly decreased (Fig. 3). The metabolite IAld was not significantly lower; however, a decreasing trend is visible. A comparison of microbial diversity also shows a significant decrease in microbial diversity in the case of newborn stool of ATB-treated mothers (Fig. 4). Treatment of mothers with ATB before or during delivery affects the concentration of metabolites and microbial diversity in the stool of newborns. The comparison of metabolite concentrations and microbiome diversity in meconium showed no significant difference.

**Fig. 5:**
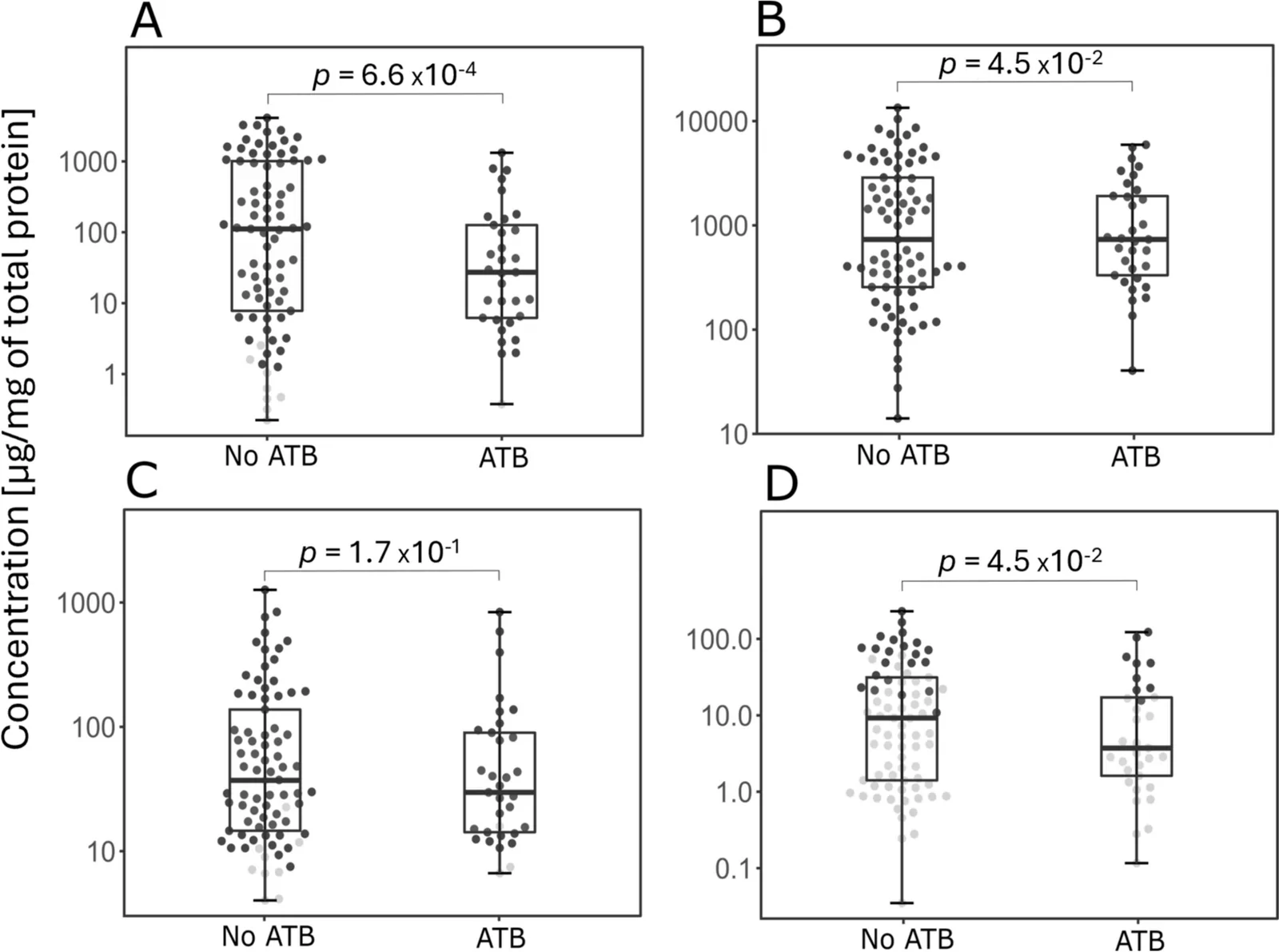
The effect of ATB-treatment of mothers on metabolite composition of stool VD newborns. Black spots indicate concentrations of metabolites. Grey spots indicate concentrations < LOQ. Boxplots indicate groups of concentration ILA (**A**), TRP (**B**), IAld (**C**), NAceTRP (**D**). Nonparametric Mann – Whitney *U* test was used here. The box represents the middle 50%, the bold horizontal line inside the box represents the median (Q2), the bottom of the box is at the first quartile (Q1; 25th percentile), the top of the box is at the third quartile (Q3; 75th percentile). Whiskers extend to the smallest and largest values within 1.5 times the IQR (1.5 × IQR) from Q1 and Q3, respectively (IQR = inter-quartile range). Points plotted individually beyond the whiskers represent outliers

**Fig. 6:**
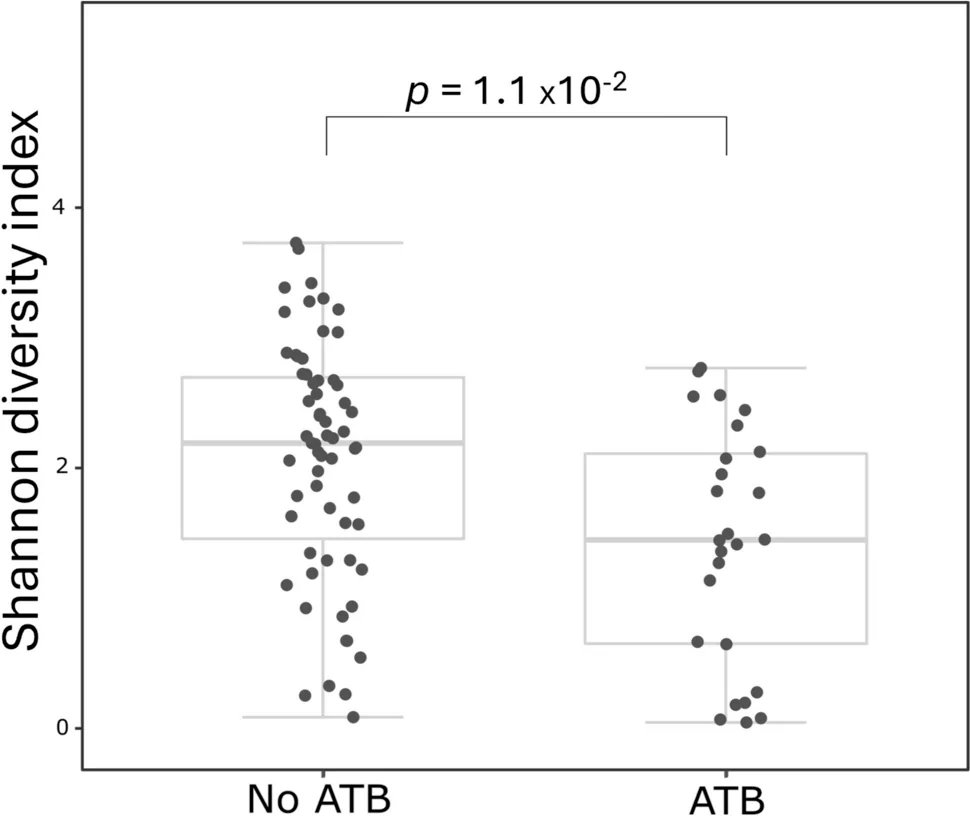
The effect of ATB-treatment of mothers on microbial diversity of stool of VD newborns. The boxplot on the left represents microbial diversity in stool samples of ATB untreated mothers. The boxplot on the right represents microbial diversity in stool samples of ATB treated mothers. The box represents the middle 50%, the bold horizontal line inside the box represents the median (Q2), the bottom of the box is at the first quartile (Q1; 25th percentile), the top of the box is at the third quartile (Q3; 75th percentile). Whiskers extend to the smallest and largest values within 1.5 times the IQR (1.5 × IQR) from Q1 and Q3, respectively (IQR = inter-quartile range). Points plotted individually beyond the whiskers represent outliers

Among 134 delivered newborns, 20 newborns were delivered by CD. Out of them, 19 mothers delivered with CD were treated with ATB. Thus, we compared the effect of ATB on metabolite and microbial diversity of VD newborn stool (Figs. 5 and 6). The concentrations of ILA, TRP and NAceTRP were significantly lower in stool of VD newborns when the mothers were treated with ATB (Fig. 5). The concentration of IAld was not significantly lower; however, the decrease is visible. Microbial diversity was significantly lower in the stool of VD newborns of ATB-treated mothers (Fig. 6). The comparison of metabolite concentrations and microbial diversity in the stool of VD and CD newborns from ATB-treated mothers did not show any significant difference (Supplementary Figure S10).

**Fig. 7:**
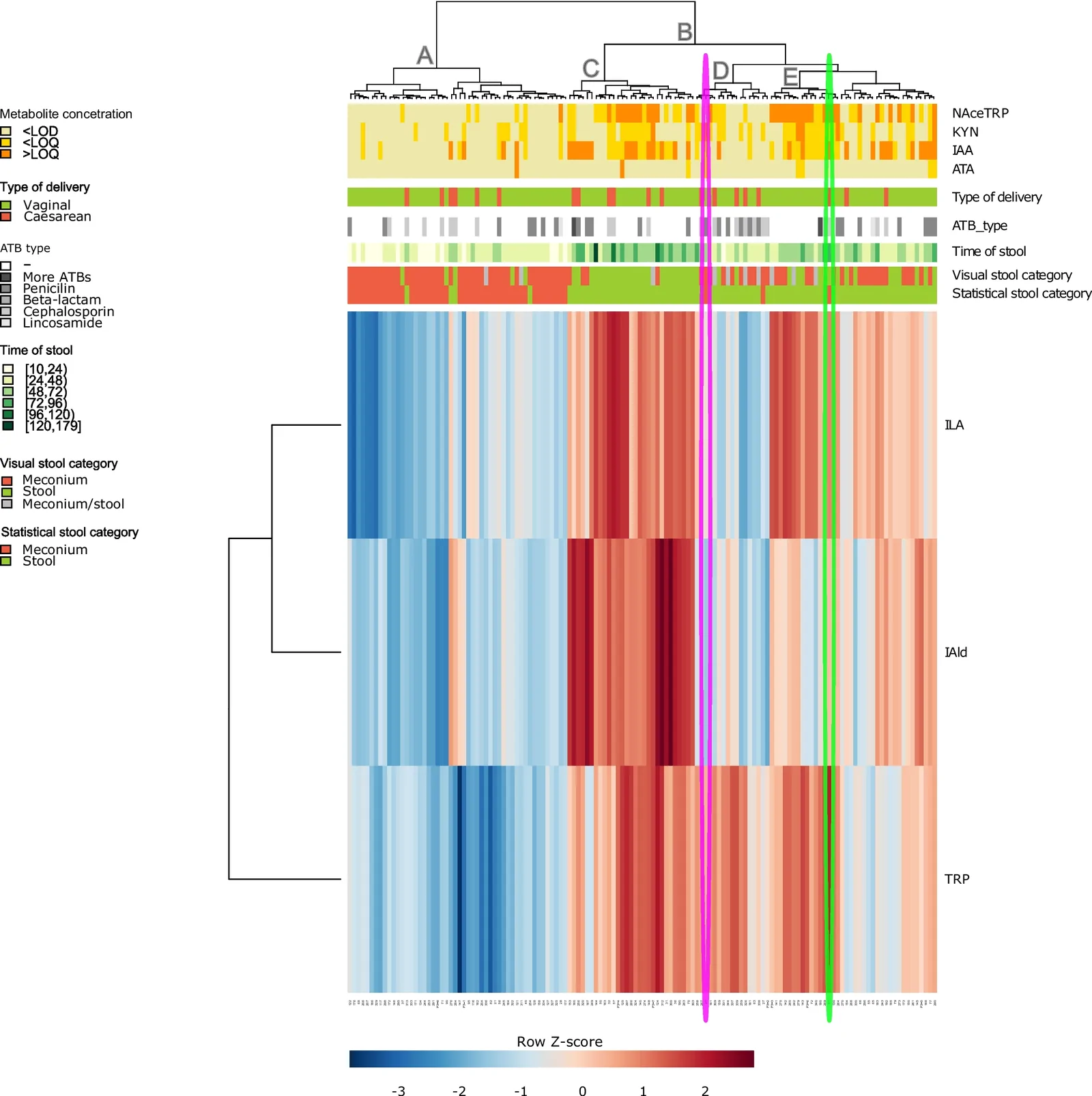
Heatmap of determined metabolites. Blue-red colour in the heatmap indicates concentrations of ILA, IAld and TRP in the sample standardized to row *Z*-score. The yellow/orange coloured plot indicates the metabolites below/above LOD/LOQ (NAceTRP, KYN, IAA, ATA). Red/green/grey coloured inserted plots indicate the type of delivery or categorization of the sample (stool, meconium). Samples were categorized based on visual identification or statistical IGHA quantification. Shades of green coloured plot indicate the hours after delivery when the sample was collected. Shades of grey coloured plots indicate ATB intake. **A**–**E** Resulting clusters. Green and pink ellipses indicate samples 67 and 25

The heatmap of metabolites shows the hierarchical clustering of all samples and three quantifiable metabolites; see Fig. 7. In cluster A (defined by low concentration of ILA, IAld and TRP), there are low concentrations of all metabolites and contains meconium samples at early time of sample collection. Cluster B contains stool samples and is split into three smaller clusters, C, D and E. Cluster C is defined by high concentrations of ILA, IAld and TRP. The concentrations of NAceTRP, KYN and IAA are also higher compared to meconium. The sample collection times are also the latest (72 h). Cluster D shows stool within 48 h and has a high concentration of TRP but low concentration of IAld, ILA, NAceTRP, KYN and IAA. The ATB-treated mothers are as well clustered. This shows that ATB usage affected the microbiome, and thus, microbial metabolism of TRP in stool. Cluster E contains stool samples with high concentrations of ILA, TRP, NAceTRP, KYN and IAA. However, the concentration of IAld is low. Comparison of clusters C and E shows that the generation of IAld is not progressed in stool samples of cluster E. Pink and green ellipse in the heatmap highlight previously mentioned samples 25 and 67. They were categorized as meconium based on the concentration of IGHA; however, metabolite PCA plot indicated possibly incorrect categorization. The position of samples 25 and 67 in the heatmap also categorizes these samples as stool.

A comparison of bacteria relative abundance in stool samples of CD and VD newborns of ATB-treated and untreated mothers is shown in Supplementary Figure S11. Comparison is shown at the phylum, class and genus levels. The bar plots show a strong similarity in bacterial abundance between stool of CD and VD newborns of mothers treated with ATB. Bacterial abundance is more diverse in the stool of VD newborns of ATB-untreated mothers.

A heatmap based on clustering of abundantly present bacteria and three quantifiable metabolites in the stool is shown in Supplementary Figure S12. Two main clusters, A and B, are indicated. Cluster A contains the most of stool samples from newborns delivered with CD and VD of ATB-treated mothers. Cluster B includes mostly stool samples from VD newborns of ATB-untreated mothers. Cluster B shows a high abundance of *Bacteroides*, *Bifidobacterium* and *Escherichia* and a higher concentration of ILA and NAceTRP, compared to cluster A. Cluster C (as a subcluster of A) has a large number of CD and had a high abundance of *Enterobacter* and *Peptoclostridium*, and IAA. Spearman correlation showed a significant positive correlation between *Bifidobacterium* and ILA (Supplementary Figure S13). *Bifidobacterium* and *Bacteroides* show a positive correlation with NAceTRP with *p* < 0.01 and *p* < 0.05. *Peptoclostridium* positively correlated with IAA with *p* < 0.001 (Supplementary Figure S13).

## Discussion

The aim of this study was to investigate the development of an early microbiome and characterize the effect of the delivery mode and ATB usage on microbial composition and, thus, the formation of TRP metabolites influencing the gut mucosal immunity of a newborn. We investigated microbiome diversity and concentration levels of microbial TRP metabolites in 134 newborn meconium and stool samples.

First, the protocol for quantification of microbial TRP metabolites in newborn meconium and stool was developed. We quantified TRP, ATA, IAA, KYN, NAceTRP, ILA and IAld in 134 meconium and stool samples. The metabolite concentrations of IAld, ILA and TRP were significantly higher in stool than in meconium. The microbiome diversity was also significantly higher in stool than in meconium. The stool microbiome is developing, and the expectation of higher microbiome diversity, thus higher concentrations of TRP metabolites, is presumed (Petersen et al. 2021). PCA plot of metabolites determined in meconium and stool and an indication of different time frames in the range of 10 to 179 h showed that time significantly affects metabolite composition during the first days of life. The metabolite composition formatted within 24 h is altered a lot after 72 h after delivery. Further, this could show a different development stage of the gut barrier between meconium and stool samples, due to different microbiome development and TRP metabolite production. PCA plot of metabolites and an indication of stool or meconium based on the concentration of IGHA showed two outliers, samples 25 and 67. Based on the concentration of IGHA, they are indicated as meconium; however, based on the TRP metabolite composition, they are more similar to stool. We assume that these samples are stool from formula-fed newborns, where breastmilk IGHA was not detected; however, TRP from the formula was metabolized.

Further, the 51 mothers were treated with beta-lactam antibiotics (ATB) or lincosamide ATB before or during delivery. Intrapartum antibiotic prophylaxis is commonly used during caesarean delivery and in group B streptococcus-positive women before vaginal delivery or due to premature rupture of membranes (Di Renzo et al. 2015). ATB affects the microbial composition of newborns (Antunes et al. 2011; Zarrinpar et al. 2018; Tapiainen et al. 2019). Our study shows that ATB treatment of mothers affects not only the microbiome diversity but also the composition and concentration of TRP metabolites in the stool of newborns. Microbial diversity and concentration of the metabolites ILA, TRP and NAceTRP were decreased in stool from newborns of ATB-treated mothers compared to those of ATB-untreated ones. Furthermore, we compared VD newborn stool only with the effect of ATB. We observed a significant decrease in microbial diversity and concentrations of TRP and TRP metabolites ILA and NAceTRP in stool of VD newborns of ATB-treated mothers compared to ATB-untreated mothers. These results show that the ATB treatment affects the microbial composition and reduces TRP and its metabolite formation. VD has a beneficial effect on the microbial development of a newborn (Kim et al. 2020). This result indicates that the benefits of VD are vanished when the mothers are treated with ATB before or during delivery. Furthermore, the 19 newborns were delivered by CD and treated with ATB right before delivery due to caesarean prophylaxis. A comparison of microbial diversity and TRP metabolite concentration in the stool of VD newborns and CD newborns, both from ATB-treated mothers, showed no difference.

These results indicate that the microbial development in VD newborns and CD newborns is similar when the mother is treated with ATB. Therefore, we compared bacterial abundance at the phylum, class and genus levels in stool samples of CD and VD newborns of ATB-treated and untreated mothers to see a difference in microbial composition. A strong similarity was observed in bacterial relative abundance between stool samples of CD and VD newborns of mothers treated with ATB. The bacterial diversity is much higher in stool samples of VD newborns of ATB-untreated mothers. This result also shows that ATB treatment reduces the bacterial diversity in CD and VD infants.

We found that a group of stool samples had a high concentration of ILA, TRP, NAceTRP, KYN and IAA. However, the concentration of IAld was low. We assume that the tryptophan side chain oxidation (TSO) pathway generation IAld is not yet fully activated, so the IAld is not formed.

The further negative effect of ATB treatment was visible in the heatmap of abundantly present bacteria and metabolites in the stool. ATB treatment of mothers caused a decrease in the abundance of the genera *Bacteroidota*, *Bifidobacterium* and *Escherichia* and concentrations of ILA and NAceTRP. The genera *Enterobacter* and *Peptoclostridium* were increased with the metabolite IAA. Similar results were found by Nogacka et al. (2017), who reported a lower proportion of *Bacteroidota* and *Actinomycetota* and an increase in *Firmicutes* and *Pseudomonadota* of ATB-treated mothers (Nogacka et al. 2017). Furthermore, stool samples from CD newborns had the highest abundance of *Enterobacter*, *Peptoclostridium* and IAA. Correlation of bacterial species abundance and concentration of metabolites showed that in the first days of life, beneficial *Bifidobacterium* could be the main producer of ILA, and *Bifidobacterium* with *Bacteroides* could be the main producers of NAceTRP. The metabolites NAceTRP and ILA are beneficial for the host’s immune system. NAceTRP was shown to be a substance P receptor antagonist and protect against neuroinflammation (Fernandes et al. 2018). ILA was shown to be produced by *Bifidobacterium* (Laursen et al. 2021) and to activate ILC3 through AhR signaling, producing IL-22 to induce resistance against mucosal candida (Zelante et al. 2013). *Peptoclostridium* strongly correlated with IAA; thus, *Peptoclostridium* could be the main producer of IAA during the first days of life. The potentially harmful bacterial genera *Enterobacter* and *Peptoclostridium* are associated with higher levels of IAA. *Enterobacter* was shown to induce spontaneous colitis (Garrett et al. 2010) and *Peptoclostridium* is a genus of bacteria, which includes the nosocomial pathogen *Peptoclostridium difficile*, which causes colitis (Wellcome Sanger Institute; Kyne et al. 2001). IAA, although it may be produced by *Enterobacter* and *Peptoclostridium*, controls inflammation by competitive inhibition of phospholipase A_2_ (Dileep et al. 2013). Although the bacterial species are potentially harmful, the tryptophan metabolite IAA has been shown to bind AhR, which, through binding, sets up an anti-inflammatory signal cascade, for example, to alleviate hepatic steatosis (Xu et al. 2021) and to reduce fatty acid and LPS production of pro-inflammatory cytokines in macrophages (Krishnan et al. 2018). We conclude that ATB treatment of mothers influenced the microbial development within the first days of the life of a newborn. The beneficial bacteria *Bifidobacterium* were suppressed with ATB. On the other hand, CD, usually accompanied by ATB treatment, caused an increased abundance of *Peptoclostridum* and *Enterobacter* and, thus, elevated concentration of IAA. Further, there could be seen a development of the metabolite level from meconium to stool as a gradual development in the gut barrier homeostasis with the metabolites IAld and ILA. The significance of our findings is in line with the fact that impaired diversity of stool microbiota can be associated with allergies, autoimmune diseases and, generally, diseases of the Western lifestyle that result from immune dysregulation. The development and regulation of the human immune system are critically dependent on mucosal microbial colonization from the first days of life. Tryptophan metabolites do not reflect the presence and activity of only one type of bacteria but the functional complexity of the entire microbiota. They can, therefore, be used as new non-invasive markers to assess the functional state and development of the gut microbiota in humans from the first days of life.

## Supplementary Information

Below is the link to the electronic supplementary material.

Supplementary file1 (PDF 1592 KB)

## Data Availability

All data on the measured metabolites in stool of newborns that support the findings of this study are included within this paper and its supplementary information files.
