# Supplementary material for: Fecal tryptophan metabolite profiling in newborns in relation to microbiota and antibiotic treatment
Source: Appl Microbiol Biotechnol. 2024 Nov 5;108:504. doi: 10.1007/s00253-024-13339-4 (PMC11538234; doi:10.1007/s00253-024-13339-4)
Supplement: Supplementary file 1 [file 253_2024_13339_MOESM1_ESM.pdf]

Applied Microbiology and Biotechnology

***Supplementary Material***

# Fecal tryptophan metabolite profiling in newborns in relation to microbiota and antibiotic treatment

**Anne-Christine Aust[1], Veronika Vidova[1], Katerina Coufalikova[1], Sona Smetanova[1], Kristyna Kozeluhova[1], Lenka Micenkova[1], Petra Videnska[1], Stanislav Smatana[1], Eva Budinska[1], Ivo Borek[2], Petr Janku[3], Jana Klanova[1], Zdenek Spacil[1], Vojtech Thon[1*]**

[1]RECETOX, Faculty of Science, Masaryk University, Brno, Czech Republic

[2]Department of Neonatology, University Hospital Brno, Brno, Czech Republic

[3]Clinic of Gynecology and Obstetrics, University Hospital Brno, Brno, Czech Republic

* Corresponding author

*** Correspondence:**

Corresponding Author: Prof. Vojtech Thon, Masaryk University, Faculty of Science, Kamenice 753/5, pavilion D29/1S101, 625 00 Brno, Czech Republic. Tel. (+420) 549 49 8043; e-mail: vojtech.thon@recetox.muni.cz

**Table of Contents**

Table S1. Characteristics of individual neonates participating in the study.

Table S2. List of primers pairs targeting the hypervariable V4 region (515F-806R) of the bacterial 16S rRNA gene.

Materials and Methods - Mass Spectrometry Data Processing.

Materials and Methods – Linearity.

Table S3. SRM assay library for positive ion detection mode. Compounds marked with a *are transitions used for quantification in the stool matrix.

Table S4. List of all metabolite standards generated for absolute quantification.

Table S5. Linear Range of calibration for isotope labelled metabolites.

Figure S1. Typical chromatograms of tryptophan catabolites in meconium and stool samples. The upper panel shows the conformity with SRM signature and retention time of measured standards, the lower panel in measured meconium and stool samples.

Figure S2. Calibration curve of Anthranilate (ATA).

Figure S3. Calibration curve of Indole-3-acetic acid (IAA).

Figure S4. Calibration curve of Kynurenine (KYN).

Figure S5. Calibration curve of Tryptophan (TRP).

Figure S6. Identification of meconium and stool samples based on the distribution of log concentration of IGHA.

Figure S7. PCA plot of metabolite (TRP, ILA and IAld) concentrations in meconium and stool– shift of metabolites composition in time after delivery.

Figure S8. Box plots of metabolites quantified in meconium and stool from 134 newborns.

Figure S9. Comparison of metabolites concentrations determined in meconium and stool samples from 134 newborns.

Figure S10. The comparison of concentrations of metabolites and microbial diversity in stool of VD and CS newborns of ATB-treated mothers.

Figure S11. Relative abundance of bacteria at various taxonomic levels in stool samples of CD and VD newborns of ATB- treated/untreated mothers.

Figure S12. Heat map of selected bacteria and metabolites in the stool.

Figure S13. Spearman correlation of TRP metabolites and microbiome abundance in stool.

**Table S1.** Characteristics of individual neonates participating in the study.

| Sample | Gestation age | Mode of delivery | Medication | Anamnesis of mother | Anamnesis of neonate | Sex | Length (cm) | Weight (g) | Apgar score |
|---|---|---|---|---|---|---|---|---|---|
| 1 | 40+2 | Vaginal | --- | --- | --- | Male | 51 | 3600 | [9, 10, 10] |
| 2 | 39+6 | Vaginal | Epidural | --- | Erythema | Male | 50 | 3240 | [10, 10, 10] |
| 3 | 40+4 | Vaginal | --- | --- | Intrauterine hypoxia, jaundice | Female | 52 | 3410 | [6, 9, 10] |
| 4 | 40+4 | Vaginal | --- | Anemia | Erythema | Male | 49 | 3240 | [9, 9, 10] |
| 5 | 41+0 | Vaginal | --- | --- | Jaundice, traumatism | Female | 50 | 3760 | [8, 9, 9] |
| 6 | 39+6 | C-Section | --- | --- | Erythema | Female | 47 | 3220 | [10, 10, 10] |
| 7 | 39+3 | Vaginal | Oxytocin | Anemia | Intrauterine hypoxia | Male | 51 | 3230 | [3, 7, 9] |
| 8 | 40+1 | Vaginal | --- | --- | --- | Male | 52 | 3630 | [10, 10, 10] |
| 9 | 40+1 | Vaginal | --- | Anemia, diabetes | --- | Female | 49 | 3320 | [10, 10, 10] |
| 10 | 39+1 | Vaginal | --- | Asthma | --- | Male | 50 | 3240 | [10, 10, 10] |
| 11 | 40+0 | Vaginal | --- | Anemia | Erythema | Female | 49 | 3820 | [10, 10, 10] |
| 12 | 41+3 | C-Section | --- | --- | Erythema | Male | 49 | 3250 | [9, 9, 10] |
| 13 | 39+3 | C-Section | --- | --- | Jaundice | Male | 46 | 2490 | [5, 8, 8] |
| 14 | 40+2 | Vaginal | Oxytocin | --- | --- | Female | 49 | 3320 | [10, 10, 10] |
| 15 | 41+0 | Vaginal | Oxytocin | *Streptococcus* | --- | Female | 49 | 3690 | [9, 10, 10] |
| 16 | 40+2 | Vaginal | Epidural | --- | Intrauterine hypoxia | Male | 50 | 3360 | [10, 10, 10] |
| 17 | 40+5 | C-Section | Epidural | Asthma | --- | Male | 51 | 3900 | [7, 7, 8] |
| 18 | 40+4 | Vaginal | --- | --- | --- | Male | 54 | 3910 | [10, 10, 10] |
| 19 | 40+5 | Vaginal | --- | Anemia, *Streptococcus* | --- | Female | 50 | 3600 | [9, 10, 10] |
| 20 | 39+5 | C-Section | --- | --- | --- | Female | 51 | 3790 | [9, 10, 10] |
| 21 | 40+0 | Vaginal | --- | --- | Intrauterine hypoxia | Male | 52 | 3800 | [10, 10, 10] |
| 22 | 38+5 | Vaginal | --- | --- | Intrauterine hypoxia, erythema | Female | 48 | 2750 | [9, 9, 9] |
| 23 | 39+3 | Vaginal | --- | --- | --- | Male | 52 | 3950 | [9, 9, 10] |

| Sample | Gestation age | Mode of delivery | Medication | Anamnesis of mother | Anamnesis of neonate | Sex | Length (cm) | Weight (g) | Apgar score |
|---|---|---|---|---|---|---|---|---|---|
| 24 | 40+0 | Vaginal | --- | --- | --- | Male | 48 | 3200 | [9, 10, 10] |
| 25 | 39+3 | Vaginal | Epidural | Anemia | Jaundice | Female | 52 | 3520 | [9, 10, 10] |
| 26 | 38+6 | Vaginal | --- | --- | Erythema, traumatism | Male | 49 | 3030 | [9, 10, 10] |
| 27 | 39+5 | Vaginal | --- | --- | --- | Male | 53 | 4100 | [10, 10, 10] |
| 28 | 39+4 | Vaginal | --- | --- | Jaundice | Female | 48 | 3040 | [10, 10, 10] |
| 29 | 40+4 | Vaginal | Epidural | --- | Intrauterine hypoxia, jaundice | Male | 51 | 3730 | [9, 10, 10] |
| 30 | 40+3 | Vaginal | Entonox | Anemia | --- | Male | 60 | 3390 | [9, 10, 10] |
| 31 | 40+3 | Vaginal | --- | Anemia | Erythema, jaundice | Male | 51 | 3770 | [9, 10, 10] |
| 32 | 39+2 | Vaginal | Epidural | Asthma | Erythema, traumatism | Male | 48 | 2960 | [10, 10, 10] |
| 33 | 39+4 | Vaginal | Epidural | --- | --- | Female | 49 | 3210 | [10, 10, 10] |
| 34 | 40+2 | Vaginal | --- | --- | --- | Male | 49 | 3370 | [9, 10, 10] |
| 35 | 40+5 | Vaginal | Oxytocin | --- | --- | Female | 49 | 3200 | [9, 10, 10] |
| 36 | 39+4 | Vaginal | --- | --- | Intrauterine hypoxia | Male | 50 | 3360 | [9, 10, 10] |
| 37 | 39+5 | Vaginal | Epidural | Anemia, *Streptococcus* | Intrauterine hypoxia | Female | 50 | 3650 | [8, 10, 10] |
| 38 | 38+6 | Vaginal | --- | Anemia | --- | Male | 49 | 3260 | [9, 10, 10] |
| 39 | 40+2 | Vaginal | --- | --- | --- | Male | 52 | 3540 | [10, 10, 10] |
| 40 | 39+6 | Vaginal | Epidural | --- | Jaundice | Female | 51 | 3550 | [9, 9, 9] |
| 41 | 40+2 | Vaginal | --- | Anemia | Congenital pneumonia | Male | 51 | 3590 | [8, 9, 9] |
| 42 | 40+2 | Vaginal | Epidural | Anemia | --- | Female | 50 | 3700 | [9, 9, 10] |
| 43 | 39+5 | Vaginal | --- | --- | Jaundice | Male | 51 | 2900 | [9, 10, 10] |
| 44 | 39+5 | Vaginal | Epidural | Asthma | Erythema, jaundice | Male | 53 | 3540 | [8, 10, 10] |
| 45 | 39+3 | C-Section | --- | *Streptococcus* | --- | Female | 50 | 2880 | [10, 10, 10] |
| 46 | 39+1 | Vaginal | --- | Asthma | Jaundice | Male | 51 | 3740 | [9, 10, 10] |
| 47 | 40+6 | Vaginal | --- | --- | Erythema | Male | 51 | 4180 | [5, 8, 9] |
| 48 | 39+0 | C-Section | Oxytocin | Anemia | --- | Female | 50 | 3000 | [10, 10, 10] |

| Sample | Gestation age | Mode of delivery | Medication | Anamnesis of mother | Anamnesis of neonate | Sex | Length (cm) | Weight (g) | Apgar score |
|---|---|---|---|---|---|---|---|---|---|
| 49 | 40+4 | Vaginal | Epidural | --- | --- | Female | 51 | 3460 | [10, 10, 10] |
| 50 | 40+1 | Vaginal | --- | --- | Traumatism | Female | 50 | 3120 | [10, 10, 10] |
| 51 | 40+0 | C-Section | --- | --- | --- | Male | 49 | 3780 | [9, 10, 10] |
| 52 | 39+3 | Vaginal | Epidural | Asthma | Erythema, traumatism | Male | 51 | 2870 | [9, 10, 10] |
| 53 | 40+3 | Vaginal | --- | Anemia | Erythema | Male | 52 | 3550 | [10, 10, 10] |
| 54 | 40+2 | Vaginal | --- | --- | Traumatism | Female | 47 | 2810 | [9, 10, 10] |
| 55 | 40+2 | Vaginal | --- | Anemia | --- | Female | 51 | 3380 | [10, 10, 10] |
| 56 | 39+6 | Vaginal | Epidural | --- | Intrauterine hypoxia, erythema | Female | 52 | 3340 | [9, 10, 10] |
| 57 | 40+4 | Vaginal | Epidural | --- | --- | Male | 55 | 4190 | [9, 10, 10] |
| 58 | 40+5 | Vaginal | Epidural | --- | --- | Female | 49 | 2670 | [10, 10, 10] |
| 59 | 40+1 | Vaginal | --- | --- | --- | Male | 52 | 3420 | [9, 10, 10] |
| 60 | 41+1 | Vaginal | --- | --- | Erythema | Female | 55 | 4290 | [9, 9, 10] |
| 61 | 40+6 | Vaginal | Epidural | *Streptococcus* | Intrauterine hypoxia | Female | 50 | 3220 | [9, 9, 10] |
| 62 | 40+3 | C-Section | Oxytocin | --- | Intrauterine hypoxia | Male | 53 | 4900 | [10, 10, 10] |
| 63 | 40+5 | Vaginal | --- | --- | --- | Male | 49 | 3300 | [10, 10, 10] |
| 64 | 38+5 | Vaginal | --- | Anemia | Jaundice | Female | 49 | 2750 | [9, 9, 10] |
| 65 | 40+6 | Vaginal | --- | --- | --- | Female | 52 | 3860 | [9, 9, 9] |
| 66 | 40+3 | Vaginal | Epidural | --- | Intrauterine hypoxia, erythema | Male | 54 | 3740 | [7, 9, 9] |
| 67 | 40+3 | Vaginal | Entonox | *Streptococcus* | --- | Male | 50 | 3030 | [10, 10, 10] |
| 68 | 39+4 | Vaginal | --- | --- | Erythema | Male | 50 | 3380 | [9, 9, 9] |
| 69 | 38+5 | C-Section | Epidural | --- | Intrauterine hypoxia, jaundice | Male | 49 | 4070 | [9, 9, 9] |
| 70 | 40+5 | Vaginal | Epidural | --- | Intrauterine hypoxia | Female | 51 | 3300 | [9, 10, 10] |
| 71 | 40+1 | Vaginal | Epidural | --- | Intrauterine hypoxia, jaundice | Male | 49 | 3280 | [9, 9, 9] |

| Sample | Gestation age | Mode of delivery | Medication | Anamnesis of mother | Anamnesis of neonate | Sex | Length (cm) | Weight (g) | Apgar score |
|---|---|---|---|---|---|---|---|---|---|
| 72 | 40+0 | Vaginal | Nalbuphine | --- | Intrauterine hypoxia, erythema | Female | 48 | 2840 | [9, 9, 10] |
| 73 | 40+6 | Vaginal | --- | --- | --- | Male | 51 | 3570 | [8, 9, 10] |
| 74 | 39+2 | Vaginal | --- | --- | --- | Male | 50 | 3150 | [10, 10, 10] |
| 75 | 41+2 | Vaginal | --- | --- | Erythema, jaundice | Male | 51 | 3580 | [5, 8, 9] |
| 76 | 40+5 | Vaginal | --- | --- | Jaundice | Female | 50 | 3120 | [5, 7, 9] |
| 77 | 40+2 | Vaginal | Oxytocin | *Streptococcus* | Erythema | Male | 52 | 4350 | [8, 9, 10] |
| 78 | 41+0 | Vaginal | Entonox | --- | --- | Male | 50 | 3420 | [9, 10, 10] |
| 79 | 40+1 | Vaginal | Oxytocin | *Streptococcus* | Intrauterine hypoxia | Male | 52 | 3290 | [5, 7, 8] |
| 80 | 40+5 | C-Section | Epidural | *Streptococcus* | Intrauterine hypoxia | Male | 52 | 3700 | [9, 10, 10] |
| 81 | 39+4 | Vaginal | --- | Anemia | Intrauterine hypoxia | Female | 50 | 4140 | [9, 10, 10] |
| 82 | 40+5 | Vaginal | Epidural | *Streptococcus* | --- | Female | 52 | 3440 | [8, 10, 10] |
| 83 | 41+1 | Vaginal | Epidural | Anemia | Traumatism | Female | 52 | 3930 | [9, 9, 10] |
| 84 | 40+5 | Vaginal | Epidural | --- | Erythema | Male | 51 | 3790 | [10, 10, 10] |
| 85 | 40+3 | Vaginal | --- | Anemia | Erythema | Female | 52 | 3760 | [10, 10, 10] |
| 86 | 40+4 | Vaginal | --- | --- | Erythema | Female | 52 | 3640 | [10, 10, 10] |
| 87 | 40+0 | Vaginal | --- | --- | Erythema | Male | 50 | 2850 | [9, 9, 10] |
| 88 | 39+2 | C-Section | --- | --- | Intrauterine hypoxia | Male | 50 | 3810 | [1, 6, 8] |
| 89 | 38+6 | Vaginal | --- | --- | --- | Male | 50 | 3530 | [9, 10, 10] |
| 90 | 38+6 | Vaginal | --- | Anemia | Erythema | Female | 50 | 3430 | [10, 10, 10] |
| 91 | 40+0 | Vaginal | --- | *Streptococcus* | --- | Female | 47 | 3230 | [10, 10, 10] |
| 92 | 40+4 | Vaginal | Epidural | --- | Erythema | Male | 52 | 3810 | [9, 10, 10] |
| 93 | 39+2 | Vaginal | --- | --- | --- | Female | 50 | 3250 | [10, 10, 10] |
| 94 | 39+2 | Vaginal | --- | --- | --- | Male | 50 | 3900 | [9, 10, 10] |
| 95 | 39+2 | C-Section | --- | --- | --- | Male | 49 | 3130 | [9, 9, 10] |
| 96 | 41+3 | Vaginal | --- | --- | --- | Female | 50 | 3700 | [9, 10, 10] |
| 97 | 38+6 | C-Section | Remifentanil | --- | Erythema, jaundice | Female | 50 | 3950 | [10, 10, 10] |

| Sample | Gestation age | Mode of delivery | Medication | Anamnesis of mother | Anamnesis of neonate | Sex | Length (cm) | Weight (g) | Apgar score |
|---|---|---|---|---|---|---|---|---|---|
| 98 | 39+3 | Vaginal | Oxytocin | --- | --- | Female | 51 | 3510 | [10, 10, 10] |
| 99 | 40+5 | Vaginal | Epidural | Anemia, diabetes | Intrauterine hypoxia | Male | 50 | 3750 | [9, 9, 9] |
| 100 | 39+6 | Vaginal | Oxytocin | --- | Jaundice | Male | 50 | 3200 | [9, 10, 10] |
| 101 | 39+3 | Vaginal | --- | --- | --- | Female | 54 | 3820 | [9, 10, 10] |
| 102 | 40+1 | Vaginal | --- | Diabetes | Intrauterine hypoxia, jaundice | Female | 51 | 4010 | [9, 10, 10] |
| 103 | 41+0 | Vaginal | --- | *Streptococcus* | Erythema | Male | 51 | 3730 | [9, 10, 10] |
| 104 | 40+2 | Vaginal | Epidural | --- | Erythema | Male | 50 | 3300 | [8, 9, 10] |
| 105 | 40+1 | Vaginal | --- | --- | Jaundice | Male | 51 | 3590 | [10, 10, 10] |
| 106 | 39+1 | C-Section | --- | Anemia | Intrauterine hypoxia | Male | 47 | 2960 | [1, 0, 0] |
| 107 | 40+2 | Vaginal | Epidural | --- | --- | Male | 50 | 3000 | [10, 10, 10] |
| 108 | 40+0 | C-Section | Epidural | --- | Erythema | Female | 47 | 2930 | [9, 10, 10] |
| 109 | 39+4 | C-Section | --- | --- | --- | Male | 47 | 3300 | [9, 10, 10] |
| 110 | 39+0 | Vaginal | --- | Anemia | Erythema | Male | 47 | 2470 | [9, 10, 10] |
| 111 | 41+3 | Vaginal | Epidural | --- | --- | Male | 51 | 4190 | [10, 10, 10] |
| 112 | 41+0 | Vaginal | --- | Asthma | --- | Female | 53 | 3550 | [8, 8, 9] |
| 113 | 39+5 | Vaginal | Oxytocin | Anemia, asthma, diabetes | --- | Female | 50 | 3730 | [9, 10, 10] |
| 114 | 39+3 | C-Section | --- | --- | --- | Male | 50 | 2960 | [9, 9, 9] |
| 115 | 41+1 | Vaginal | --- | Anemia | Intrauterine hypoxia | Male | 52 | 3170 | [8, 10, 10] |
| 116 | 40+3 | Vaginal | --- | Diabetes | --- | Female | 51 | 3680 | [9, 10, 10] |
| 117 | 40+4 | Vaginal | --- | --- | --- | Male | 50 | 4130 | [10, 10, 10] |
| 118 | 40+4 | Vaginal | --- | Anemia | Erythema | Female | 51 | 4110 | [9, 9, 10] |
| 119 | 39+2 | Vaginal | --- | --- | --- | Male | 48 | 3180 | [9, 10, 10] |
| 120 | 40+0 | Vaginal | --- | *Streptococcus* | --- | Female | 51 | 3820 | [9, 9, 9] |
| 121 | 40+6 | Vaginal | --- | --- | --- | Female | 53 | 4450 | [9, 10, 10] |
| 122 | 39+0 | Vaginal | --- | --- | --- | Male | 48 | 3230 | [9, 9, 9] |

| Sample | Gestation age | Mode of delivery | Medication | Anamnesis of mother | Anamnesis of neonate | Sex | Length (cm) | Weight (g) | Apgar score |
|---|---|---|---|---|---|---|---|---|---|
| 123 | 38+2 | C-Section | Epidural | Anemia | --- | Male | 49 | 3230 | [9, 10, 10] |
| 124 | 41+1 | C-Section | --- | --- | --- | Female | 53 | 3930 | [10, 10, 10] |
| 125 | 40+2 | Vaginal | --- | --- | --- | Male | 51 | 4000 | [10, 10, 10] |
| 126 | 40+6 | Vaginal | --- | --- | Erythema, jaundice | Male | 53 | 4130 | [7, 9, 10] |
| 127 | 40+1 | Vaginal | --- | --- | --- | Female | 52 | 3620 | [9, 9, 10] |
| 128 | 39+4 | Vaginal | --- | --- | Jaundice | Male | 49 | 3450 | [7, 8, 9] |
| 129 | 40+6 | Vaginal | Epidural | --- | Jaundice, traumatism | Female | 52 | 3690 | [10, 10, 10] |
| 130 | 40+2 | Vaginal | Entonox | --- | --- | Male | 50 | 3380 | [9, 9, 9] |
| 131 | 40+4 | Vaginal | Oxytocin | --- | Intrauterine hypoxia | Male | 54 | 4560 | [9, 10, 10] |
| 132 | 39+6 | Vaginal | Oxytocin | Anemia | Erythema, jaundice, traumatism | Male | 50 | 3090 | [9, 10, 10] |
| 133 | 39+5 | Vaginal | --- | --- | Erythema | Female | 49 | 2980 | [9, 10, 10] |
| 134 | 40+1 | Vaginal | --- | Diabetes | Intrauterine hypoxia, jaundice | Male | 52 | 3430 | [10, 10, 10] |

**Table S2.** List of primer pairs targeting the hypervariable V4 region (515F-806R) of the bacterial 16S rRNA gene

| Primer name | | 5´-sequence-3´ | Reference of primers |
|---|---|---|---|
| **EMP16S-1** | F | TCGTCGGCAGCGTCAGATGTGTATAAGAGACAGAGCCTTCGTCGCGTGTGYCAGCMGCCGCGGTAA | **16S Metagenomic Sequencing Library protocol**; Illumina, San Diego, California, USA (EMP515-806) |
| | R | GTCTCGTGGGCTCGGAGATGTGTATAAGAGACAGCCTAACGGTCCACCGGACTACNVGGGTWTCTAAT | |
| **EMP16S-2** | F | TCGTCGGCAGCGTCAGATGTGTATAAGAGACAGTCCATACCGGAAGTGTGYCAGCMGCCGCGGTAA | |
| | R | GTCTCGTGGGCTCGGAGATGTGTATAAGAGACAGCGCGCCTTAAACCCGGACTACNVGGGTWTCTAAT | |
| **EMP16S-3** | F | TCGTCGGCAGCGTCAGATGTGTATAAGAGACAGAGCCCTGCTACAGTGTGYCAGCMGCCGCGGTAA | |
| | R | GTCTCGTGGGCTCGGAGATGTGTATAAGAGACAGTATGGTACCCAGCCGGACTACNVGGGTWTCTAAT | |
| **EMP16S-4** | F | TCGTCGGCAGCGTCAGATGTGTATAAGAGACAGTGAGACCCTACAGTGTGYCAGCMGCCGCGGTAA | |
| | R | GTCTCGTGGGCTCGGAGATGTGTATAAGAGACAGGCCTCTACGTCGCCGGACTACNVGGGTWTCTAAT | |
| **EMP16S-5** | F | TCGTCGGCAGCGTCAGATGTGTATAAGAGACAGACTTGGTGTAAGGTGTGYCAGCMGCCGCGGTAA | |
| | R | GTCTCGTGGGCTCGGAGATGTGTATAAGAGACAGACTACTGAGGATCCGGACTACNVGGGTWTCTAAT | |
| **EMP16S-6** | F | TCGTCGGCAGCGTCAGATGTGTATAAGAGACAGATTACGTATCATGTGTGYCAGCMGCCGCGGTAA | |
| | R | GTCTCGTGGGCTCGGAGATGTGTATAAGAGACAGAATTCACCTCCTCCGGACTACNVGGGTWTCTAAT | |
| **EMP16S-7** | F | TCGTCGGCAGCGTCAGATGTGTATAAGAGACAGCACGCAGTCTACGTGTGYCAGCMGCCGCGGTAA | |
| | R | GTCTCGTGGGCTCGGAGATGTGTATAAGAGACAGCGTATAAATGCGCCGGACTACNVGGGTWTCTAAT | |
| **EMP16S-8** | F | TCGTCGGCAGCGTCAGATGTGTATAAGAGACAGTGTGCACGCCATGTGTGYCAGCMGCCGCGGTAA | |
| | R | GTCTCGTGGGCTCGGAGATGTGTATAAGAGACAGATGCTGCAACACCCGGACTACNVGGGTWTCTAAT | |
| **EMP16S-9** | F | TCGTCGGCAGCGTCAGATGTGTATAAGAGACAGCCGGACAAGAAGGTGTGYCAGCMGCCGCGGTAA | |
| | R | GTCTCGTGGGCTCGGAGATGTGTATAAGAGACAGACTCGCTCGCTGCCGGACTACNVGGGTWTCTAAT | |
| **EMP16S-10** | F | TCGTCGGCAGCGTCAGATGTGTATAAGAGACAGTTGCTGGACGCTGTGTGYCAGCMGCCGCGGTAA | |
| | R | GTCTCGTGGGCTCGGAGATGTGTATAAGAGACAGTTCCTTAGTAGTCCGGACTACNVGGGTWTCTAAT | |
| **EMP16S-11** | F | TCGTCGGCAGCGTCAGATGTGTATAAGAGACAGTACTAACGCGGTGTGTGYCAGCMGCCGCGGTAA | |
| | R | GTCTCGTGGGCTCGGAGATGTGTATAAGAGACAGCGTCCGTATGAACCGGACTACNVGGGTWTCTAAT | |
| **EMP16S-12** | F | TCGTCGGCAGCGTCAGATGTGTATAAGAGACAGGCGATCACACCTGTGTGYCAGCMGCCGCGGTAA | |
| | R | GTCTCGTGGGCTCGGAGATGTGTATAAGAGACAGACGTGAGGAACGCCGGACTACNVGGGTWTCTAAT | |
| **EMP16S-13** | F | TCGTCGGCAGCGTCAGATGTGTATAAGAGACAGCAAACGCACTAAGTGTGYCAGCMGCCGCGGTAA | |
| | R | GTCTCGTGGGCTCGGAGATGTGTATAAGAGACAGGGTTGCCCTGTACCGGACTACNVGGGTWTCTAAT | |
| **EMP16S-14** | F | TCGTCGGCAGCGTCAGATGTGTATAAGAGACAGGAAGAGGGTTGAGTGTGYCAGCMGCCGCGGTAA | |
| | R | GTCTCGTGGGCTCGGAGATGTGTATAAGAGACAGCATATAGCCCGACCGGACTACNVGGGTWTCTAAT | |
| **EMP16S-15** | F | TCGTCGGCAGCGTCAGATGTGTATAAGAGACAGTGAGTGGTCTGTGTGTGYCAGCMGCCGCGGTAA | |
| | R | GTCTCGTGGGCTCGGAGATGTGTATAAGAGACAGGCCTATGAGATCCCGGACTACNVGGGTWTCTAAT | |

**Mass Spectrometry Data Processing.** The area under a chromatographic peak (AUC) was used to calculate concentration (nM and mg/L). The AUC and the known concentration of labelled internal standards were used to calculate the LOD (Equation 2) and LOQ (Equation 3) using the slope of the calibration curve.

$$LOD = \frac{SD * 3}{slope}$$

*Equation S-1: Calculation for the Limit of Detection for labelled standard.*

$$LOQ = \frac{SD * 10}{slope}$$

*Equation S-2: Calculation of the Limit of Quantification for labelled standards.*

For non-labelled standards, the response factor determined for each analyte was used to calculate respective LOD (Equation 4) and LOQ (Equation 5).

$$LOD = \frac{LOD_{labelled\ metabolite}}{RF_{labelled\ metabolite}}$$

*Equation S-3: Calculation of Limit of Detection for non-labelled metabolites.*

$$LOQ = \frac{LOQ_{labelled\ metabolite}}{RF_{labelled\ metabolite}}$$

*Equation S-4: Calculation of Limit of Quantification for non-labelled metabolites.*

**Linearity.** The measured metabolites were normalized through the ratio between an unlabelled and a labelled metabolite peak area. The normalized metabolite area was plotted against the metabolite concentration of non-labelled metabolites. The area measured for each dilution was plotted against the concentration of the non-labelled metabolite. For every labelled standard in the linearity curve, the Coefficient of variation (CV) was calculated (Equation 6).

$$\text{Coefficient of Variation} = \frac{\bar{x}_{Area\ of\ sample}\ x\ 100}{s_{Standard\ deviation\ of\ each\ dilution\ step}}$$

*Equation S-5: Calculation of Coefficient of Variation (CV)*

**Table S3.** SRM assay library for positive ion detection mode. Compounds marked with a *are transitions used for quantification in the stool matrix.

| **Compound** | **Precursor m/z** | **Product m/z** | **RT [min]** |
|---|---|---|---|
| L-Kynurenine* | 209.09 | 192 | 1.6 |
| L-Kynurenine | 209.09 | 94 | 1.6 |
| [$^{2}D_{4}$] L-Kynurenine* | 215.13 | 198 | 1.6 |
| [$^{2}D_{4}$] L-Kynurenine | 215.13 | 96.1 | 1.6 |
| L-Tryptophan | 205.1 | 188 | 2.9 |
| L-Tryptophan* | 205.1 | 91 | 2.9 |
| [$^{13}C_{11}$] [$^{15}N_{2}$] L-Tryptophan | 218.1 | 200 | 2.9 |
| [$^{13}C_{11}$] [$^{15}N_{2}$] L-tryptophan* | 218.1 | 98.1 | 2.9 |
| Anthranilic acid | 138.06 | 120 | 7.2 |
| Anthranilic acid* | 138.06 | 65 | 7.2 |
| [$^{13}C_{6}$] Anthranilic acid | 144.08 | 126.1 | 7.2 |
| [$^{13}C_{6}$] Anthranilic acid* | 144.08 | 70.2 | 7.2 |
| *N*-Acetyl-tryptophan* | 247.11 | 188.1 | 7.8 |
| *N*-Acetyl-tryptophan | 247.11 | 130 | 7.8 |
| *N*-Acetyl-tryptophan | 247.11 | 118.1 | 7.8 |
| Indole-3-lactic acid | 206.06 | 188 | 7.8 |
| Indole-3-lactic acid | 206.06 | 130.1 | 7.8 |
| Indole-3-lactid acid* | 206.06 | 117.9 | 7.8 |
| Indole-3-aldehyde* | 146.06 | 118.1 | 8 |
| Indole-3-aldehyde | 146.06 | 117.1 | 8 |
| Indole-3-aldehyde | 146.06 | 91.1 | 8 |
| Indole-3-aldehyde | 146.06 | 65.1 | 8 |
| Indole-3-aldehyde | 146.06 | 39.2 | 8 |

**Table S4.** List of all metabolite standards generated for absolute quantification.

| Analyte | Short name | Average molecular weight (MW) | Spiked metabolite concentration (nM) | Quantified selected reaction monitoring (SRM) transition | Retention time (min) | Calibration | | |
|---|---|---|---|---|---|---|---|---|
| | | | | | | LOD (nmol/L) | LOQ (nmol/L) | $R^2$ |
| Anthranilate | ATA | 137.1 | 50 | 138.1 → 120 | 7.1 | 3 | 8 | 0.9997 |
| Indole-3-acetic acid | IAA | 175.2 | 200 | 176.1 → 130.1 | 8.2 | 20 | 67 | 0.9993 |
| Indole-3-aldehyde | IAld | 145.2 | 500 | 146.1 → 118.1 | 8.1 | 18 | 62 | - |
| Indole-3-lactic acid | ILA | 205.2 | 500 | 206.1 → 117.9 | 7.9 | 2 | 8 | - |
| L-Kynurenine | KYN | 208.2 | 50 | 209.1 → 192 | 1.6 | 2 | 8 | 0.999 |
| *N*-Acetyl-tryptophan | NAceTRP | 246.3 | 500 | 247.1 → 159 | 8.2 | 25 | 83 | - |
| Tryptophan | TRP | 204.2 | 1000 | 205.1 → 188 | 2.9 | 10 | 16 | 0.9999 |

**Table S5**. Linear calibration range [nM] for isotope labelled metabolites

| Compound | Linear calibration range [nM] |
|---|---|
| Anthranilate | 8 - 500 |
| Indole-3-acetic acid | 67 - 1000 |
| Kynurenine | 8 - 250 |
| Tryptophan | 16 - 10000 |

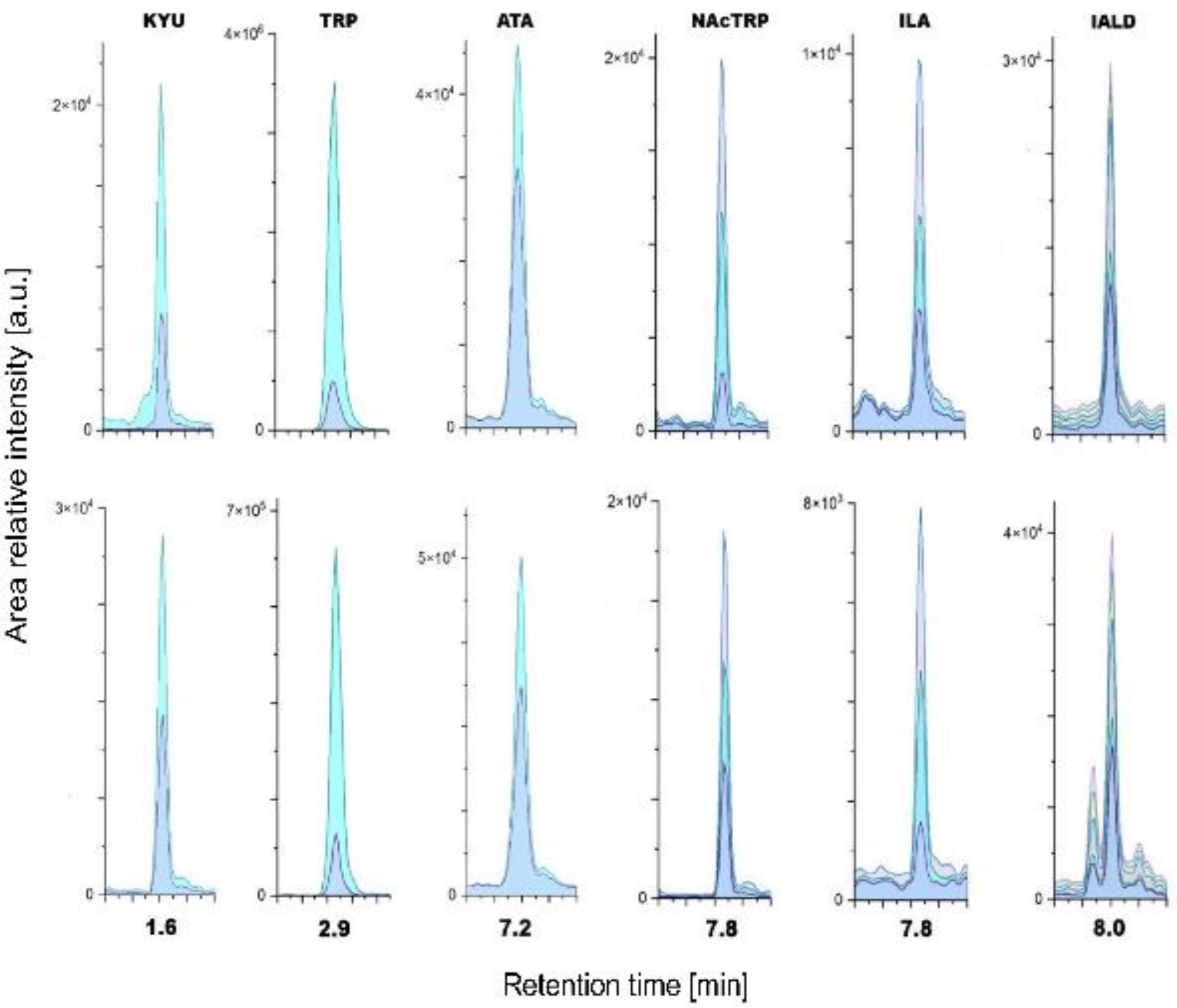


**Figure S1:** Typical chromatograms of tryptophan catabolites in meconium and stool samples. The upper panel shows the conformity with SRM signature and retention time of measured standards, the lower panel in measured meconium and stool samples. The relative intensity is given as Area relative intensity [a.u.], a.u. = arbitrary units.

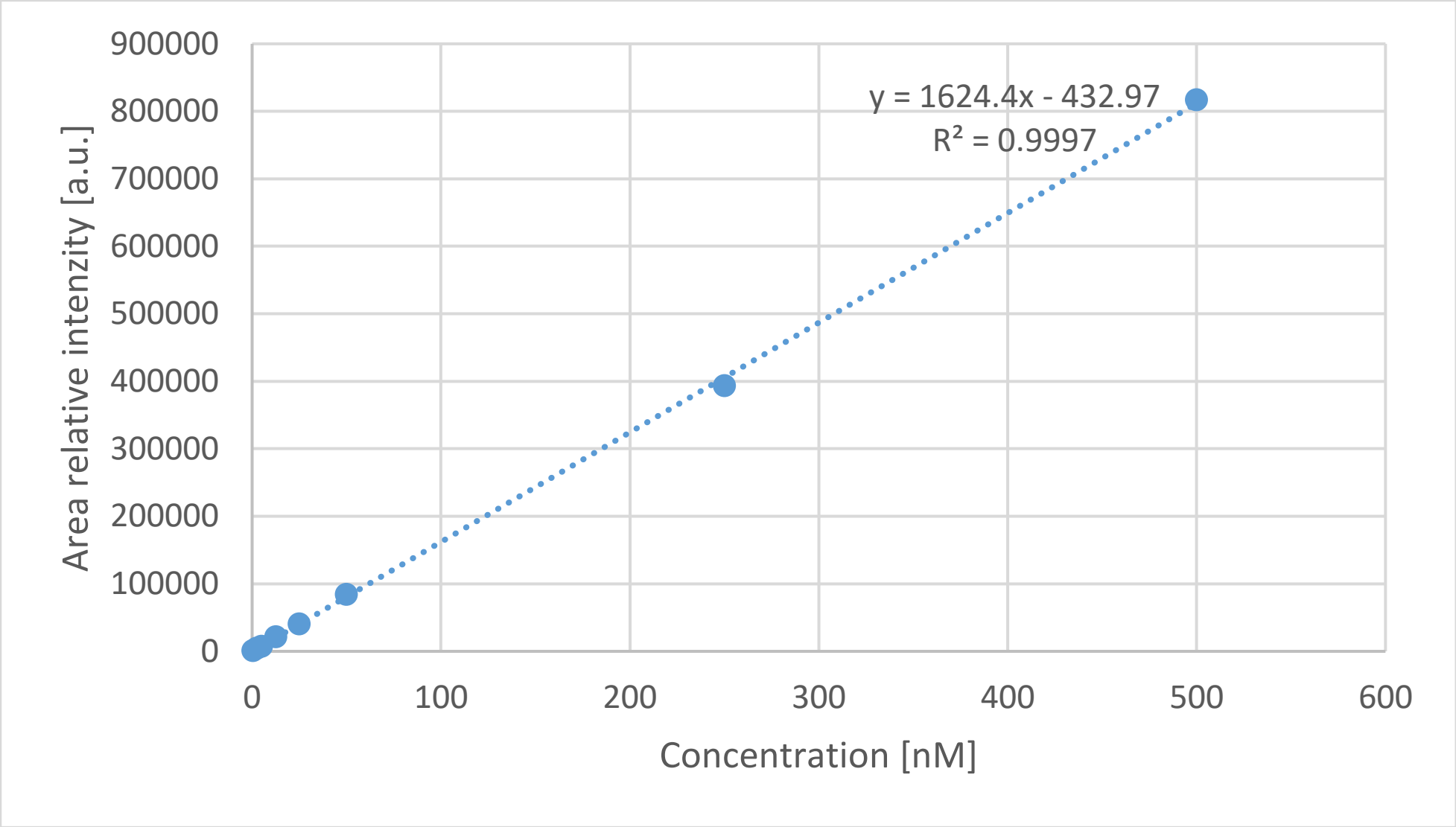


**Figure S2:** Calibration curve of ATA.

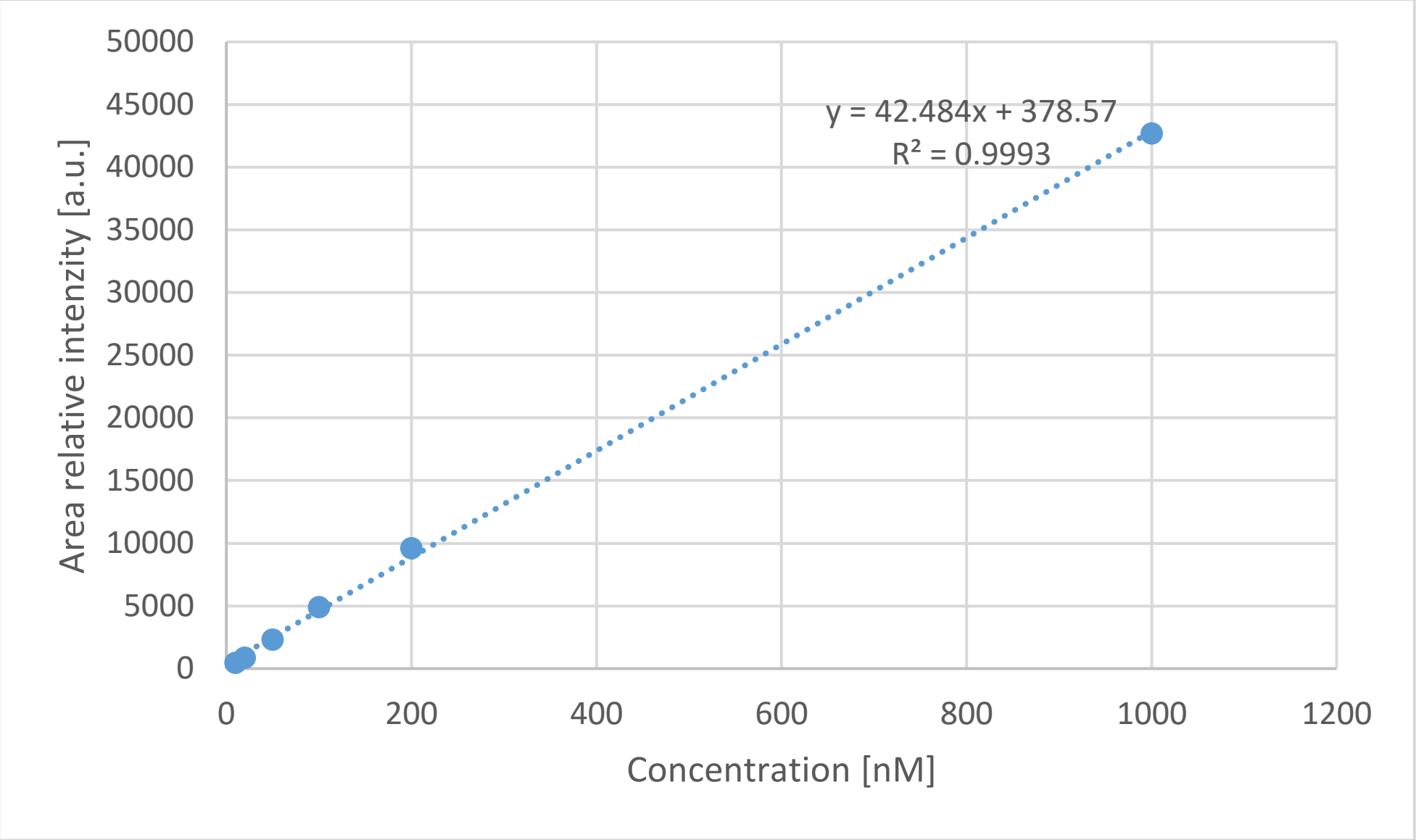


**Figure S3:** Calibration curve of IAA.

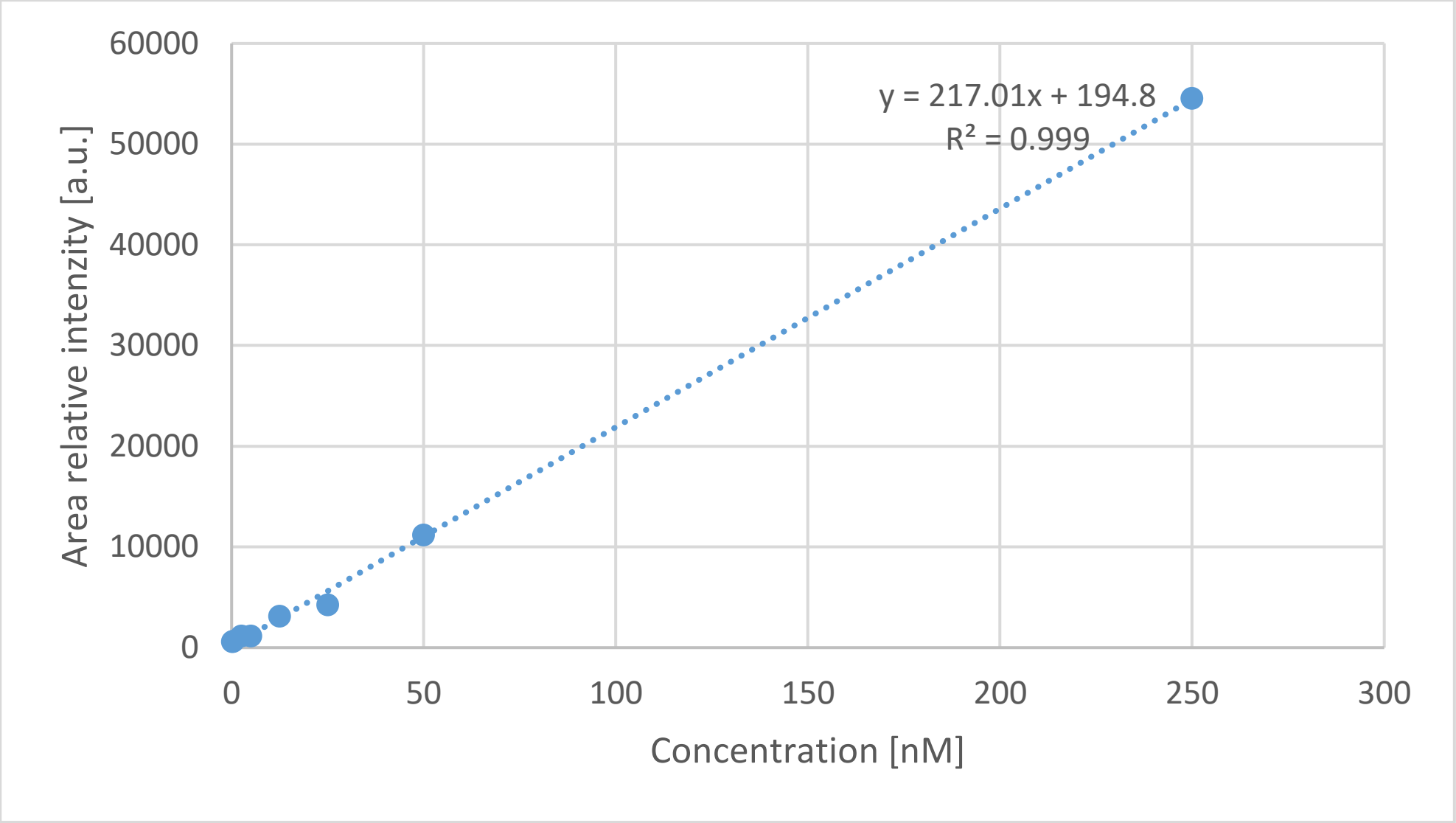


**Figure S4:** Calibration curve of KYN.

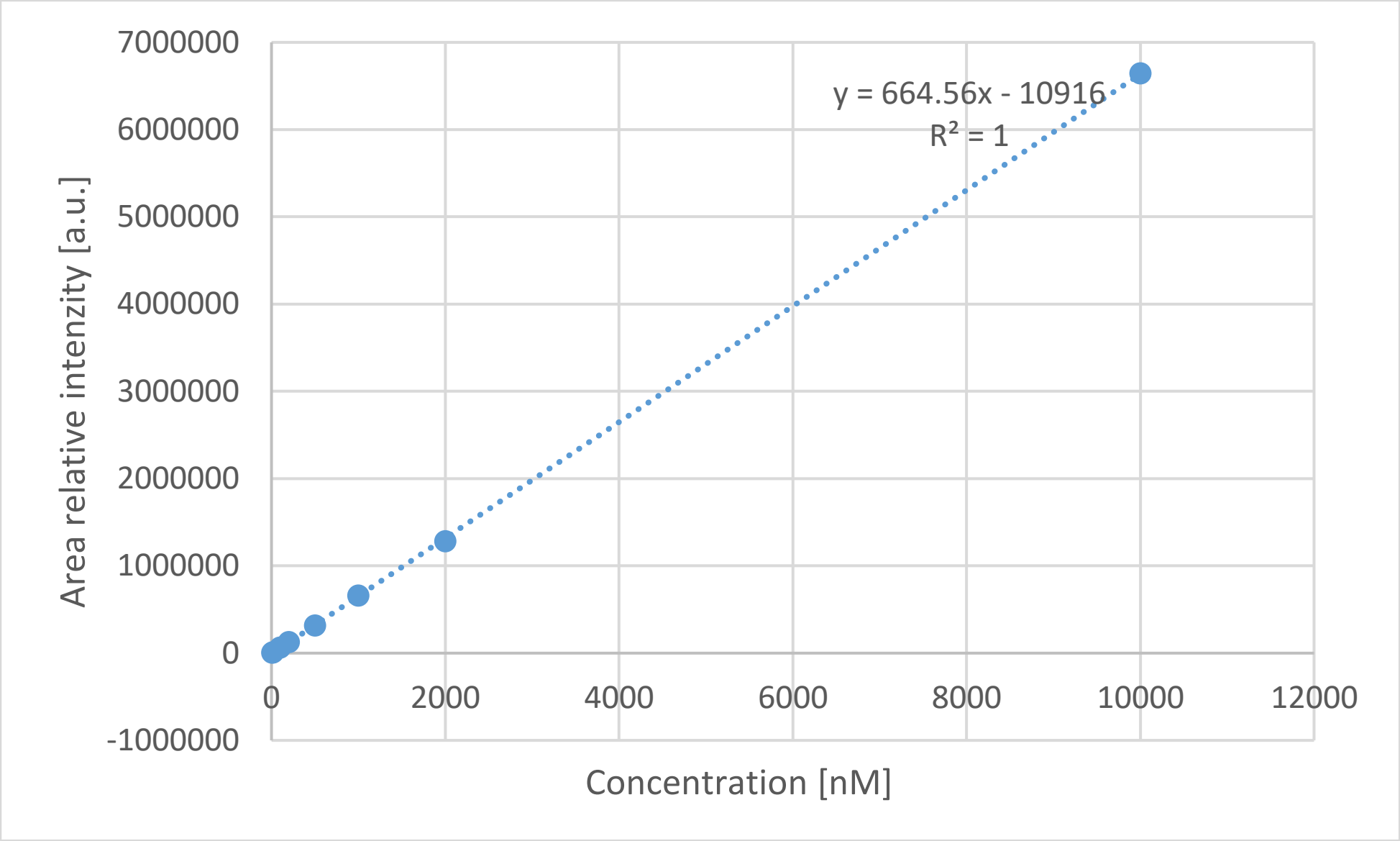


**Figure S5:** Calibration curve of TRP.

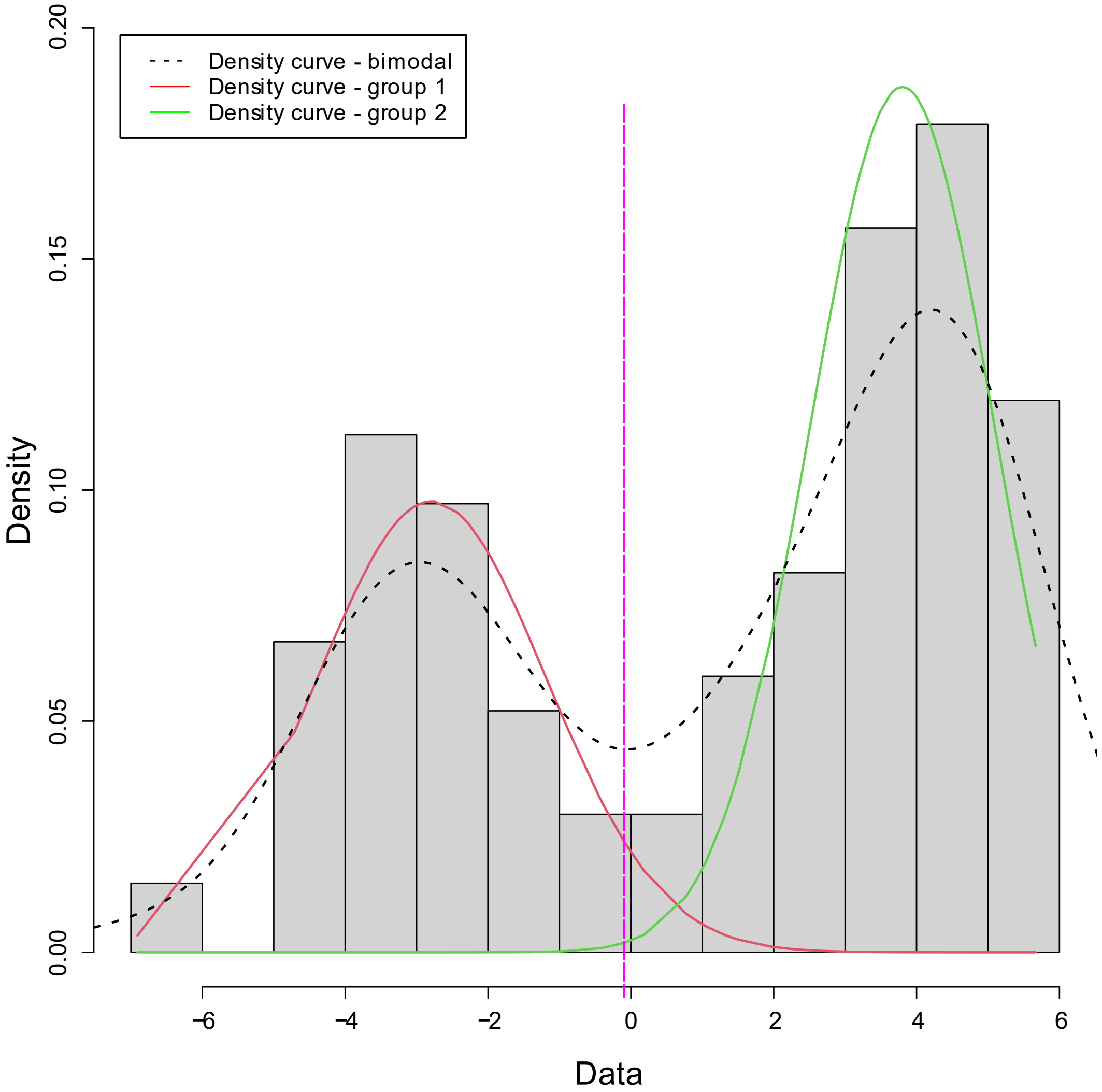


**Figure S6:** Identification of meconium and stool samples based on distribution of log concentration of IGHA. Red density curve represents distribution of samples determined as meconium. Green density curve represents the distribution of samples determined as stool. Dashed curve shows the bimodal distribution. Purple line indicates the local minimum used for the threshold to define stool or meconium.

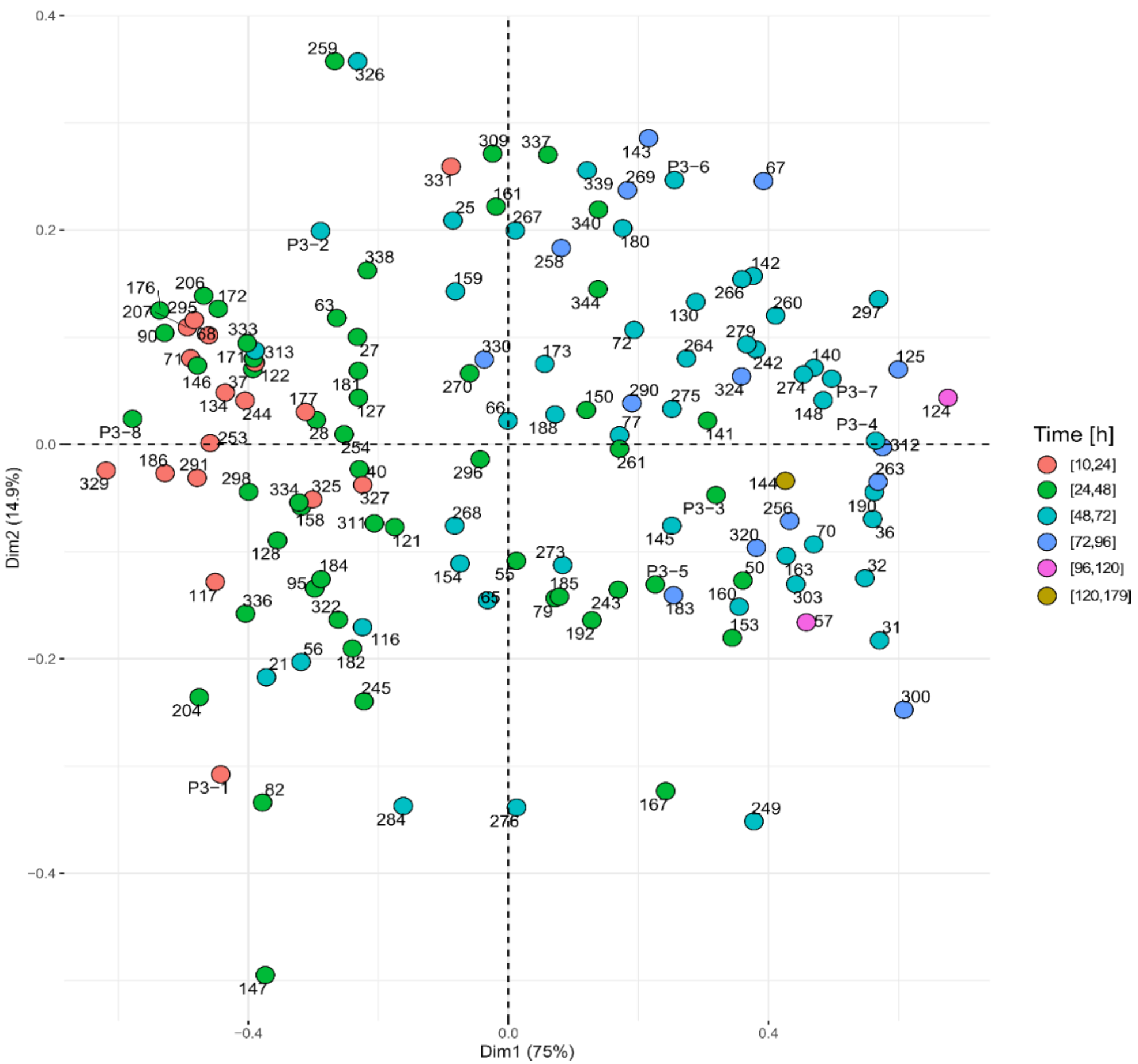


**Figure S7:** PCA plot of metabolites (TRP, ILA and IAld) concentrations in meconium and stool samples – conversion of metabolites composition in time after delivery. Red dots represent meconium or stool passed by within 24 hours, green 24 – 48 hours, turquoise 48 – 72 hours, blue 72 – 96 hours, pink 96 to 120 hours and brown 120 hours and brown 120 – 179 hours after delivery.

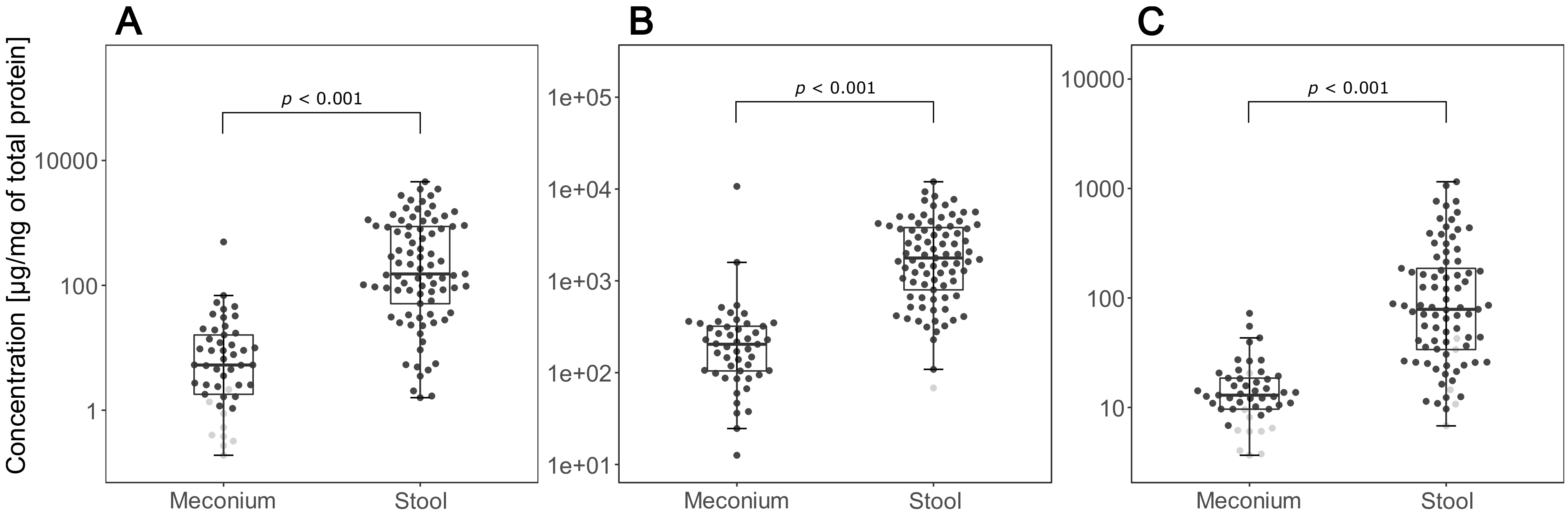


**Figure S8:** Box plots of metabolites quantified in meconium and stool of 134 newborns. Determined statistical difference of ILA (A), TRP (B) and IAld (C) in meconium and stool. Grey spots indicate values <LOQ. The box represents the middle 50%, the bold horizontal line inside the box represents the median (Q2), the bottom of the box is at the first quartile (Q1; 25th percentile), the top of the box is at the third quartile (Q3; 75$^{th}$ percentile). Whiskers extend to the smallest and largest values within 1.5 times the IQR (1.5 * IQR) from Q1 and Q3, respectively (IQR = inter-quartile range). Points plotted individually beyond the whiskers represent outliers.

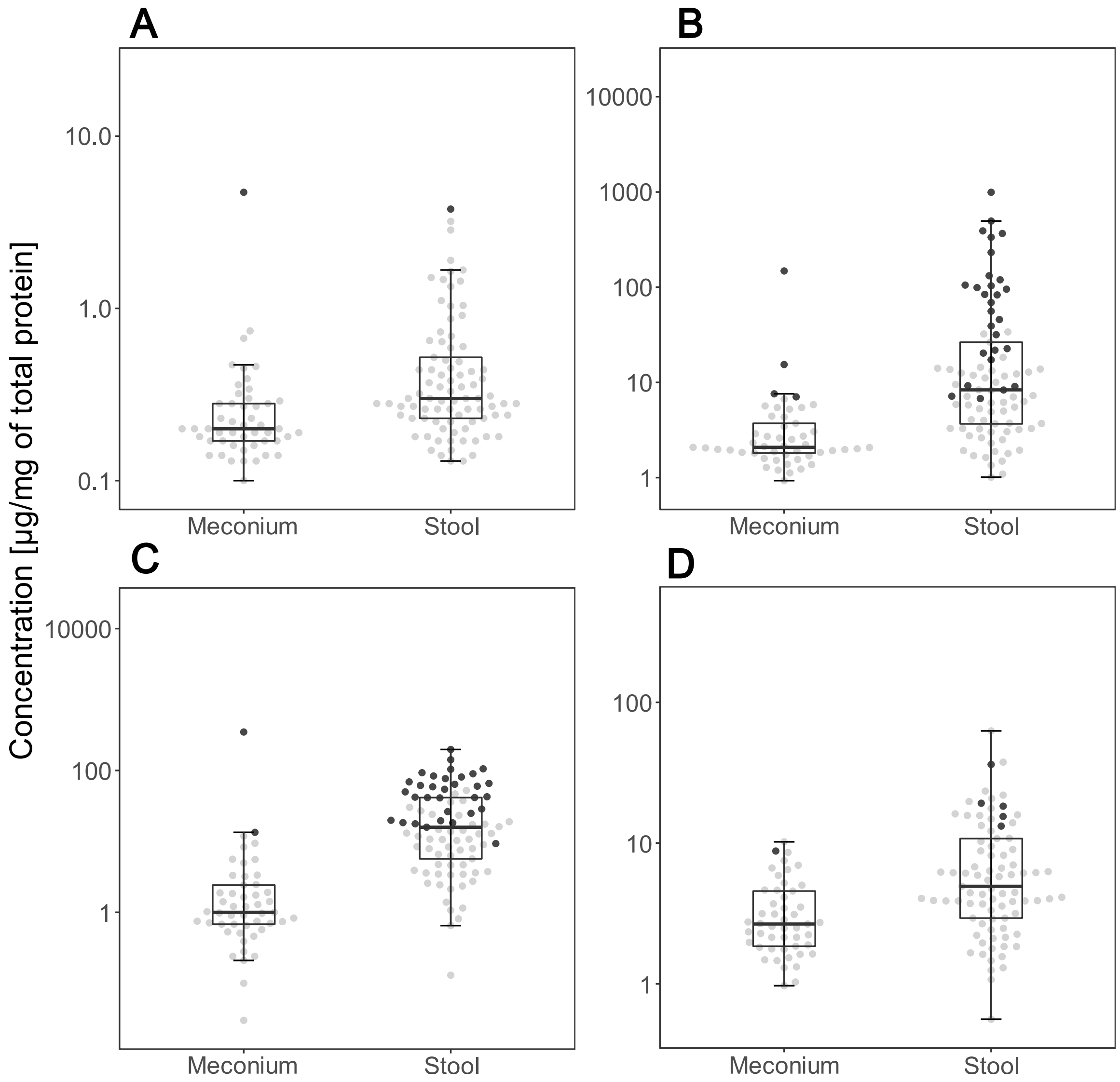


**Figure S9:** Comparison of metabolites concentrations determined in meconium and stool samples from 134 newborns. Box plots represent groups of concentrations of ATA (A), IAA (B), NAceTRP (C) and KYN (D). Black spots represent quantified values. Grey spots represent values <LOQ.

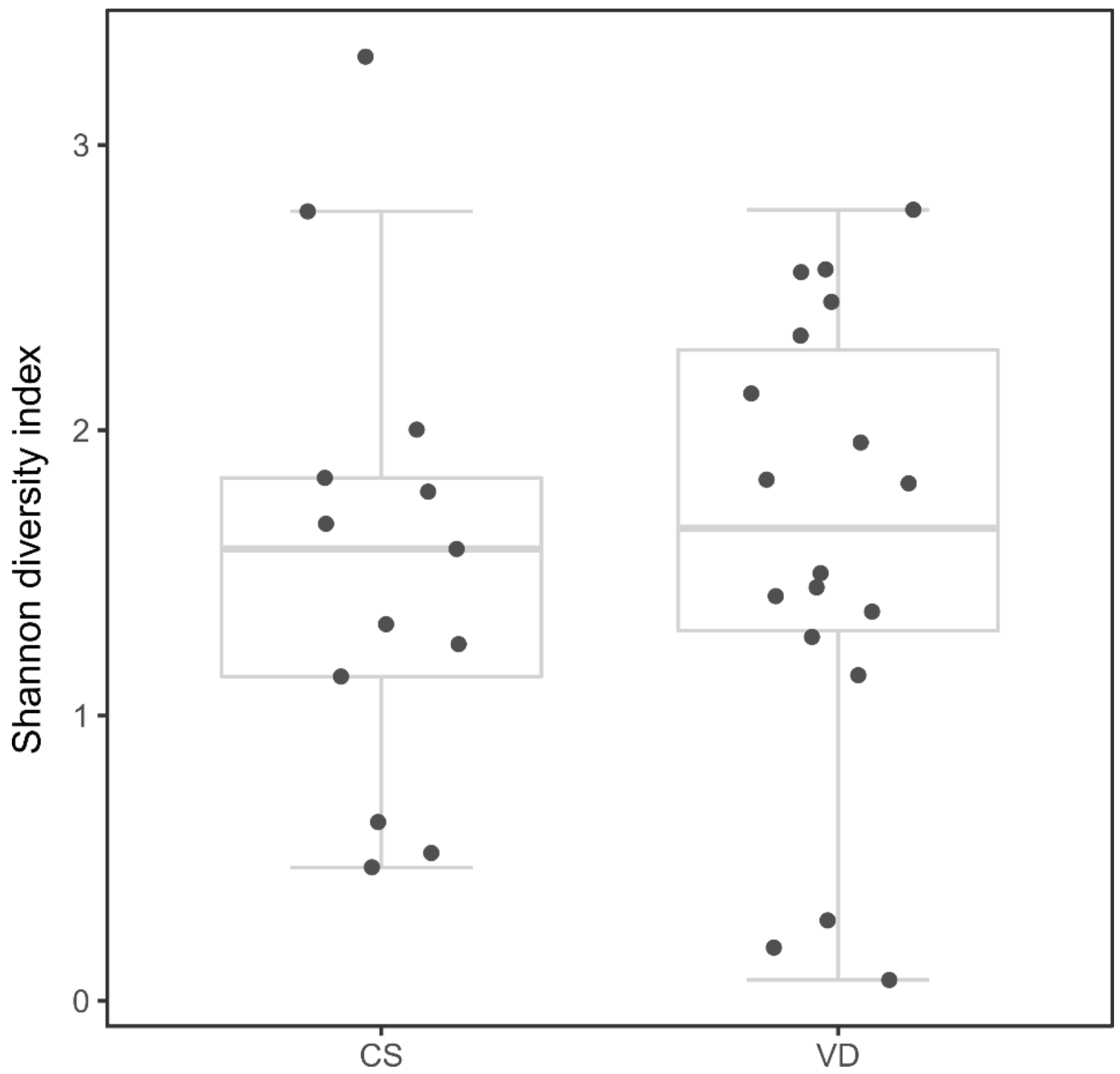


**Figure S10:** The comparison of concentrations of microbial diversity in stool of VD and CS newborns of ATB-treated mothers. The box represents the middle 50%, the bold horizontal line inside the box represents the median (Q2), the bottom of the box is at the first quartile (Q1; 25th percentile), the top of the box is at the third quartile (Q3; 75$^{th}$ percentile). Whiskers extend to the smallest and largest values within 1.5 times the IQR (1.5 * IQR) from Q1 and Q3, respectively (IQR = inter-quartile range). Points plotted individually beyond the whiskers represent outliers.

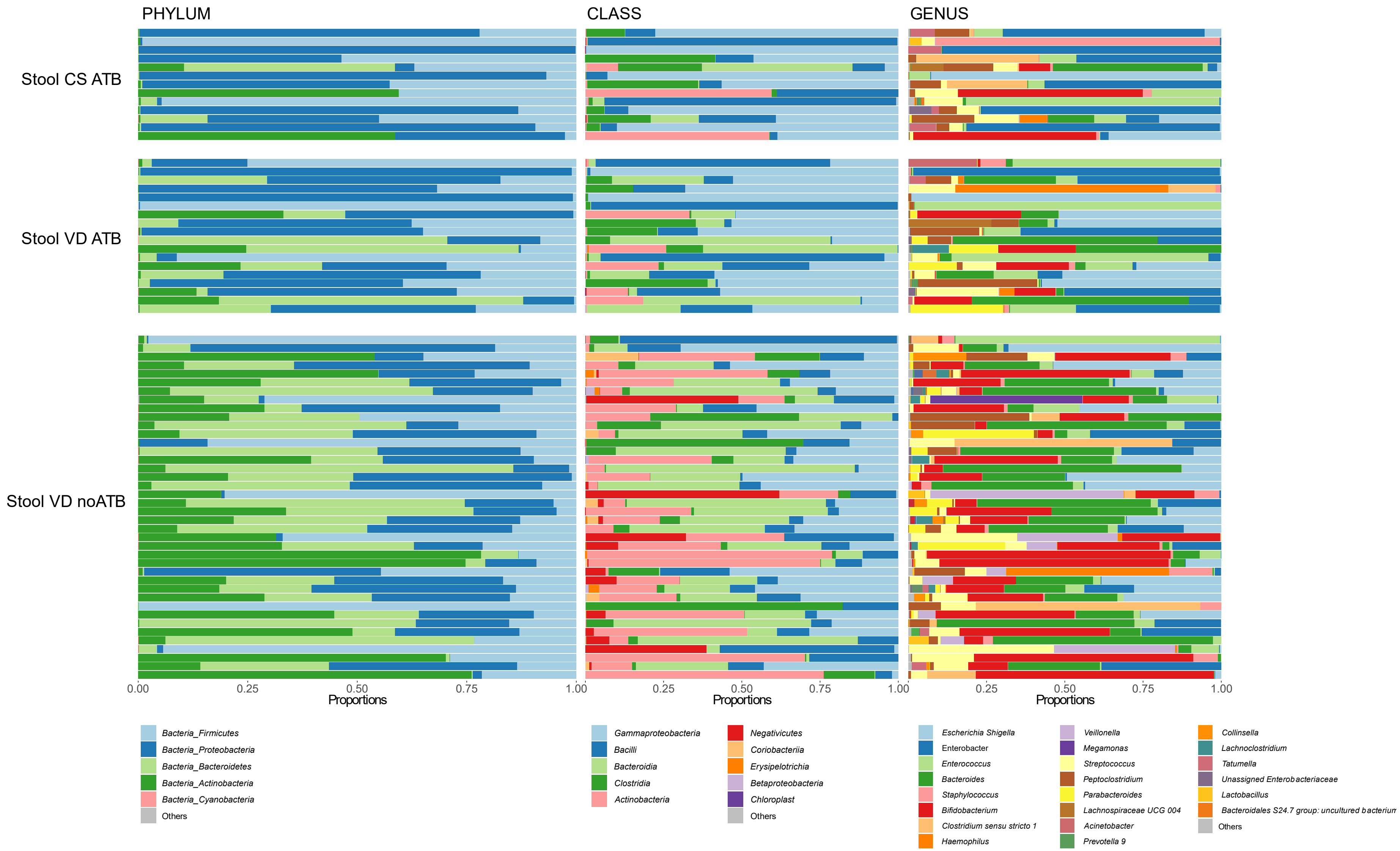


**Figure S11:** Relative abundance of bacteria at various taxonomic levels in stool samples of CS and VD newborns of ATB-treated/untreated mothers. The “others” means the sum of taxa with relative abundance in the sample < 0.01.

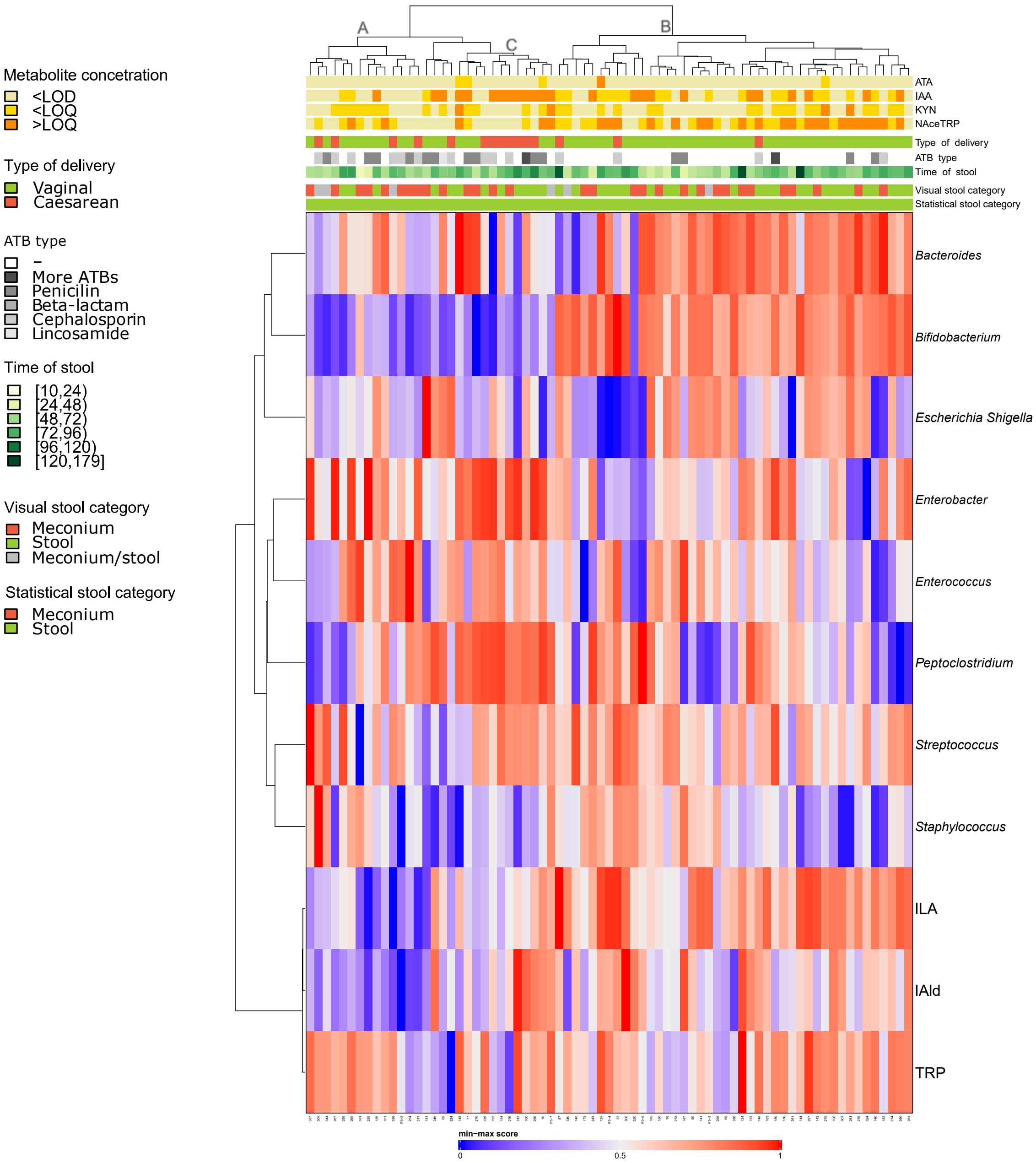


**Figure S12:** Heat map of selected bacteria and metabolites in stool. The darker the red/blue colour, the higher is amount of bacteria o concentration of ILA, IAld and TRP in the sample. The yellow/orange coloured plot indicates the metabolites below/above LOD/LOQ (NAceTRP, KYN, IAA, ATA). Red/green/grey coloured inserted plots indicate type of delivery or categorization of sample based on visual identification. Shades of green coloured plot indicate the hours when sample was collected after delivery. Shades of grey coloured plots indicate ATB intake. Letters A, B and C indicate resulting clusters.

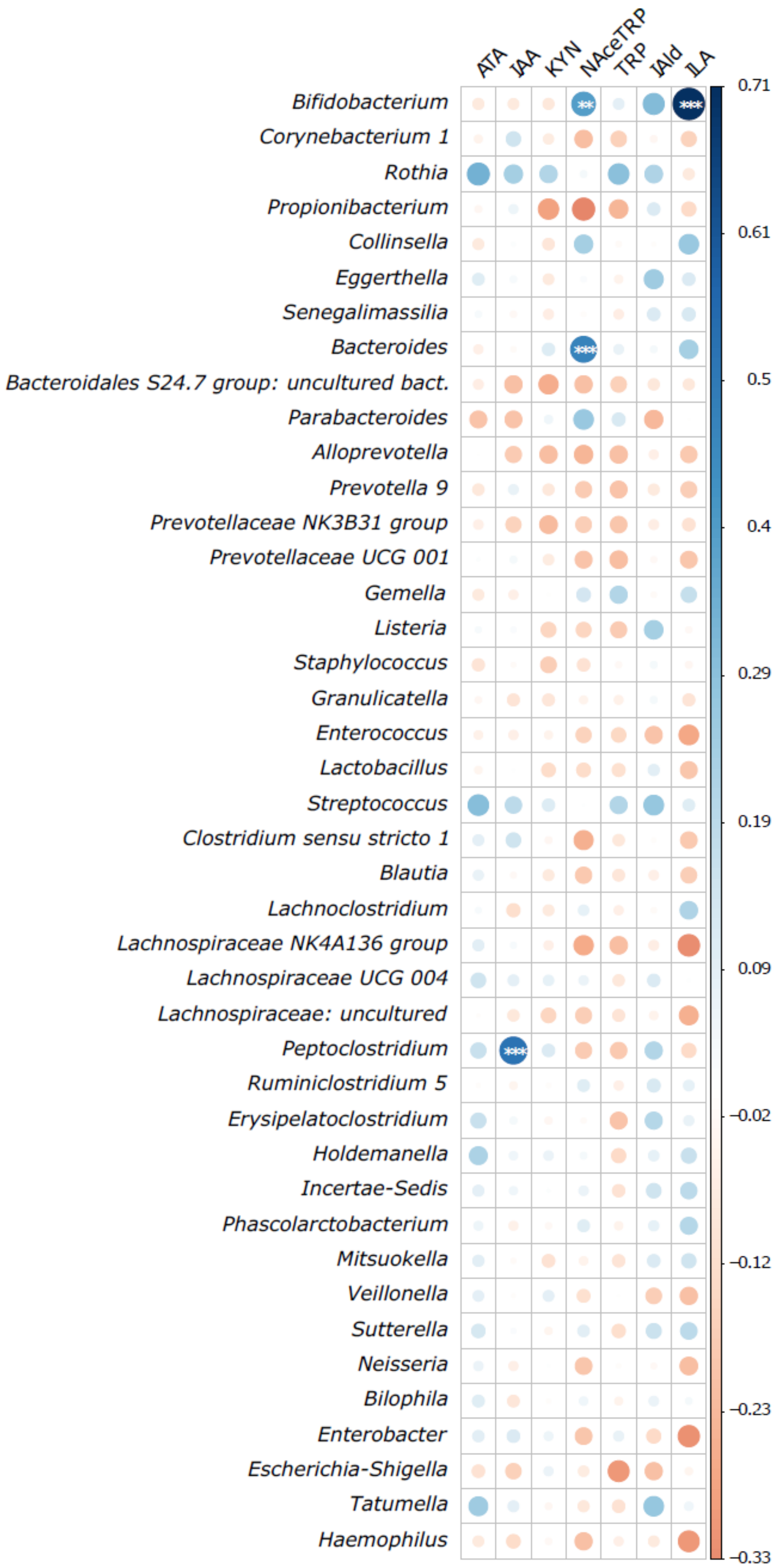


**Figure S13:** Spearman correlation of TRP metabolites and microbiome abundance in the stool. The colour indicates Spearman correlation coefficients. Statistical significance is marked with * for FDR<0.1, ** for FDR<0.05 and *** for FDR<0.01.
